# Extracellular Matrix Remodeling Alleviates Memory Deficits in Alzheimer's Disease by Enhancing the Astrocytic Autophagy‐Lysosome Pathway

**DOI:** 10.1002/advs.202400480

**Published:** 2024-06-17

**Authors:** Qinghu Yang, Chengxiang Yan, Yahan Sun, Zhen Xie, Liang Yang, Ming Jiang, Junjun Ni, Beining Chen, Sen Xu, Zhaoyue Yuan, Yanyan Wu, Xia Liu, Zengqiang Yuan, Zhantao Bai

**Affiliations:** ^1^ School of Life Science & Research Center for Natural Peptide Drugs, Shaanxi Engineering & Technological Research Centre for Conservation & Utilization of Regional Biological Resources Yanan University Yanan 716000 China; ^2^ Yanan Engineering & Technological Research Centre for Resource Peptide Drugs, Yanan Key Laboratory for Neural Immuno‐Tumor and Stem Cell Yanan 716000 China; ^3^ The Brain Science Center Beijing Institute of Basic Medical Sciences Beijing 100850 China; ^4^ Key Laboratory of Molecular Medicine and Biotherapy Department of Biology School of Life Science Beijing Institute of Technology Beijing 100081 China; ^5^ State Key Laboratory of Reproductive Medicine, Key Laboratory of Human Functional Genomics of Jiangsu Province, Department of Neurobiology, Interdisciplinary InnoCenter for Organoids, School of Basic Medical Sciences Nanjing Medical University Nanjing 211166 China

**Keywords:** Alzheimer's disease, astrocyte, autophagy‐lysosome, extracellular matrix remodeling

## Abstract

Extracellular matrix (ECM) remodeling is strongly linked to Alzheimer's disease (AD) risk; however, the underlying mechanisms are not fully understood. Here, it is found that the injection of chondroitinase ABC (ChABC), mimicking ECM remodeling, into the medial prefrontal cortex (mPFC) reversed short‐term memory loss and reduced amyloid‐beta (Aβ) deposition in 5xFAD mice. ECM remodeling also reactivated astrocytes, reduced the levels of aggrecan in Aβ plaques, and enhanced astrocyte recruitment to surrounding plaques. Importantly, ECM remodeling enhanced the autophagy‐lysosome pathway in astrocytes, thereby mediating Aβ clearance and alleviating AD pathology. ECM remodeling also promoted Aβ plaque phagocytosis by astrocytes by activating the astrocytic phagocytosis receptor MERTK and promoting astrocytic vesicle circulation. The study identified a cellular mechanism in which ECM remodeling activates the astrocytic autophagy‐lysosomal pathway and alleviates AD pathology. Targeting ECM remodeling may represent a potential therapeutic strategy for AD and serve as a reference for the treatment of this disease.

## Graphical abstract

ECM remodeling reversed short‐term memory deficits and decreased Aβ deposition in AD mice. Additionally, ECM remodeling reactivated astrocytes and facilitated their recruitment to neighboring plaques, thereby promoting Aβ clearance. Notably, ECM remodeling enhanced the autophagy‐lysosome pathway, activated the phagocytic receptors, and augmented the vesicle recycling in astrocytes. This facilitated Aβ removal of and alleviated AD‐associated pathology.

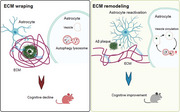

## 1 Introduction

Alzheimer's disease (AD) is the most commonly diagnosed form of dementia and is characterized by amyloid beta (Aβ) accumulation, the presence of neurofibrillary tangles, cognitive impairment, and memory loss. Recent studies have reported that reduced synapse density, neuronal loss, glial cell activation, and extracellular matrix (ECM) network disruption are hallmarks of AD.^[^ ^1^, ^2^ ^]^ The ECM refers to a large, rigid, non‐cellular structure secreted and assembled by a variety of cell types (neurons, oligodendrocytes, astrocytes, and microglia, among others) that wraps around cell somata, axon initial segments, and synapses. The ECM provides structural support for cellular and tissue organizational integrity and resilience and is a dynamic structure that is constantly remodeled for the control of organizational homeostasis.^[^ ^3^, ^4^ ^]^ Mounting evidence suggests that ECM components colocalize with senile plaques and neurofibrillary tangles.^[^ ^5^, ^6^, ^7^ ^]^ Fibronectin and hyaluronan, core structural components of the ECM, are upregulated in patients with AD and mouse models of the disease.^[^ ^8^, ^9^, ^10^ ^]^ Conversely, aggrecan (ACAN), another vital component of the brain's ECM, has been reported to exhibit both upregulation and downregulation in AD patients and AD model mice.^[^ ^10^, ^11^ ^]^

The ECM plays a vital role in the etiology of AD. In a recent review, Rahman and Lendel reported that several of the many ECM components found in plaques appear to be actively involved in the initiation and buildup of Aβ aggregates.^[^ ^7^ ^]^ The loss of perineuronal nets (PNN), ECM structures that wrap around neurons and can protect them from the neurotoxic effects of Aβ,^[^ ^2^ ^]^ is a salient phenotype in both AD patients and 5xFAD mice.^[^ ^12^ ^]^ It has been demonstrated that remodeling the GAG chain to modify PNN structure using chondroitinase ABC (ChABC) significantly enhances synaptic plasticity and improves learning and memory ability.^[^ ^13^ ^]^ Treatment with soluble collagen VI, an ECM core protein, blocks the association of Aβ oligomers with neurons, augments Aβ aggregation, and prevents neurotoxicity.^[^ ^14^ ^]^ Additionally, enhancing fibronectin‐domain III containing 5/irisin levels rescues synapse plasticity and memory defects in APPswe/PS1ΔE9 mice.^[^ ^15^, ^16^ ^]^ Furthermore, the application of heparan sulfate proteoglycans, the primary ECM skeleton molecules, was reported to decrease peripheral Aβ_1−42_ flow to the brain and increase Aβ efflux from the brain, thereby reducing the deposition of Aβ in the brain and improving cognitive defects in APPswe/PS1ΔE9 mice.^[^ ^17^ ^]^ The maintenance of ECM homeostasis, also named ECM remodeling, exhibits a strong correlation with AD pathology, and a comprehensive understanding of this homeostasis may unveil strategies for delaying the onset of AD pathology, as well as facilitate the discovery of novel therapeutics for AD.

ECM components are synthesized and modified within encapsulated cells, particularly astrocytes, before being secreted from the cell to form the ECM network. Astrocytes can synthesize ECM proteoglycans, including those of the lectican family, tenascin C, and chondroitin sulfate proteoglycans (CSPGs), as well as ECM‐degrading enzymes, such as matrix metalloproteinases (MMPs) and a disintegrin and metalloproteinase with thrombospondin motifs (ADAMTS) family members.^[^ ^18^, ^19^ ^]^ These astrocyte‐derived molecules may contribute to ECM remodeling both in physiological states and under pathological conditions that affect the brain.^[^ ^20^ ^]^ Additionally, it has been reported that ECM remodeling can direct autophagy in a molecular and cell context‐specific manner.^[^ ^21^, ^22^ ^]^ Collectively, these studies have implicated ECM remodeling in AD pathogenesis; however, the precise mechanisms by which ECM remodeling modulates the pathogenesis of AD and the consequent cognitive deficits remain poorly understood.

Here, we provide evidence that ChABC promotes the expression of ECM remodeling‐related enzymes and alters the expression of ECM components, resulting in the remodeling of the ECM in the prefrontal cortex (PFC) of AD model mice. ECM remodeling reversed the loss of short‐term memory and decreased Aβ deposition in 5xFAD mice. Additionally, ECM remodeling reactivated astrocytes and facilitated their recruitment to neighboring plaques, thereby promoting Aβ clearance. Notably, ECM remodeling enhanced the autophagy‐lysosome pathway in astrocytes, thus facilitating the removal of Aβ and alleviating AD‐associated pathology. Finally, ECM remodeling activated the phagocytic receptor and augmented the vesicle recycling process in astrocytes located in the medial PFC (mPFC) of AD mice. Our findings indicated that ECM remodeling induces the activation of the astrocytic autophagy‐lysosomal pathway, thereby alleviating AD pathology.

## 2 Results

### 2.1 ChABC Promoted ECM Remodeling in the mPFC of AD Model Mice

**Figure 1:**
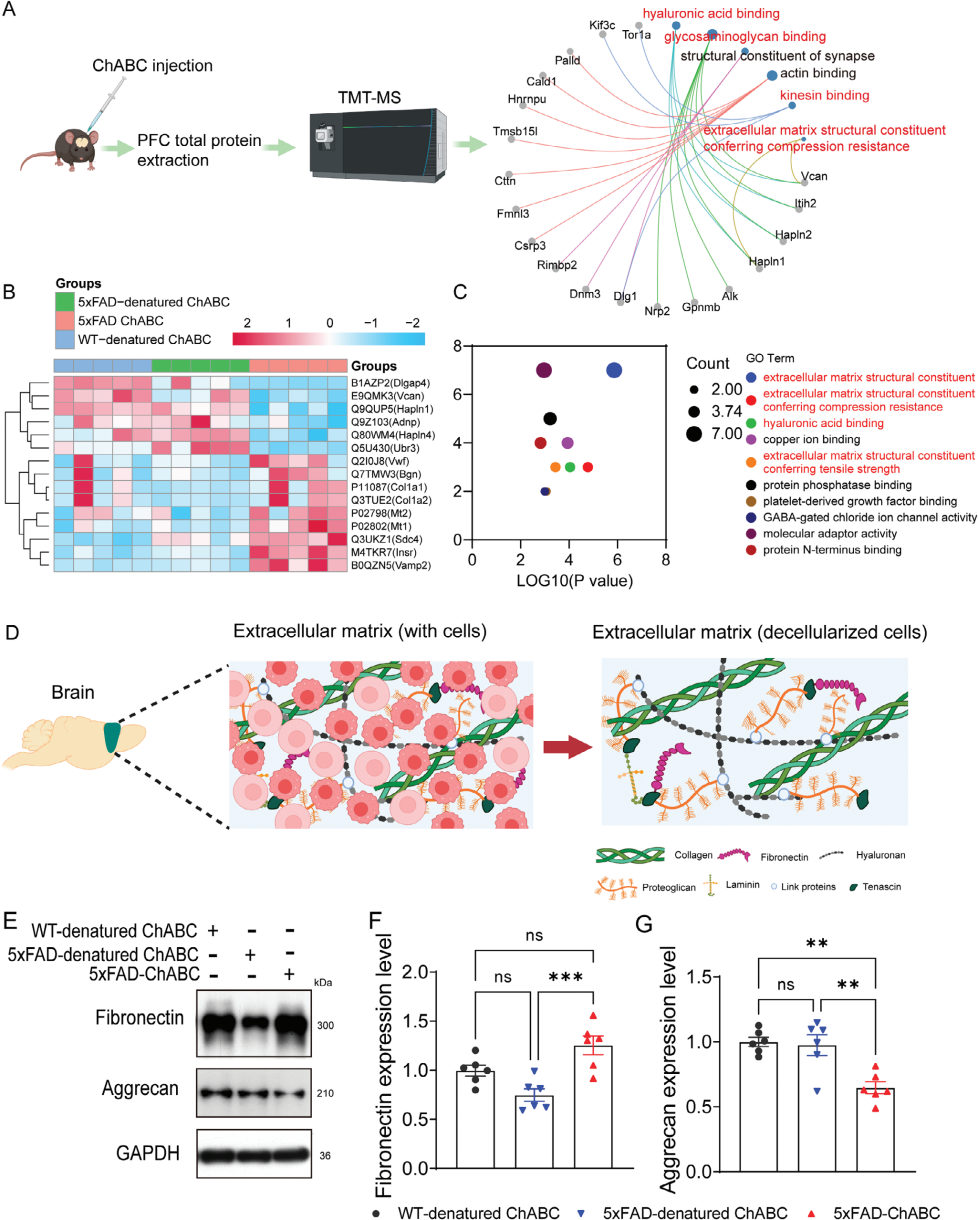
ChABC promotes extracellular matrix remodeling in the mPFC of AD model mice. A) Left: schematic of experiment. Right: The significantly enriched Gene Ontology (GO) terms of differentially expressed proteins screened by proteomics in WT mice after ChABC and denatured ChABC administration. B) Heatmap shows the top 15 differentially expressed proteins screened by proteomics after ChABC and denatured ChABC administration. C) Bubble plot of significantly enriched top 10 Gene Ontology (GO) terms of differentially expressed proteins screened by proteomics in 5xFAD mice after ChABC and denatured ChABC administration. D) Schematic diagram of the brain cell decellularization process. E) Western blotting analysis of fibronectin and aggrecan after decellularization in PFC from WT‐denatured ChABC, 5xFAD‐denatured ChABC, and 5xFAD ChABC mice. F,G) Quantification of the expression level of fibronectin (F) and aggrecan(G) in the western blotting shown in (E). Data in (F) and (G) are means ± SEM (numbers in bars show biological replicates). (A) – (C) have five biological replicates. Statistical analyses were performed with One‐way ANOVA with Bonferroni's multiple comparisons test, *P < 0.05, **P < 0.01, ns, no significant. For additional data, see Figure S1–S3 (Supporting Information).

ChABC, a chondroitin sulfate hydrolyzing enzyme found in the ECM,^[^ ^23^ ^]^ represents a powerful tool for investigating the composition and functionality of the ECM. Following the application of ChABC in the mPFC of wild‐type (WT) mice, total protein was extracted from the mPFC and subjected to Tandem Mass Tag–Mass Spectrometric (TMT**–**MS) analysis. The identified differentially expressed proteins were primarily associated with hyaluronic acid binding, glycosaminoglycan binding, kinesin binding, and extracellular matrix structural constituent conferring compression resistance (**Figure** **1**A; Data S1, Supporting Information), suggesting that the administration of ChABC may potentially alter the composition of the ECM, a process commonly referred to as ECM remodeling.^[^ ^4^ ^]^ Additionally, we performed RNA sequencing on PFC tissues from both WT and AD mice, with the results showing that 453 genes were upregulated and 442 were downregulated in the PFC of AD mice compared with that in WT mice (Figure S1A, Supporting Information). These differentially expressed genes (DEGs) were mainly associated with the immune process, glia activation, cytokine and chemokine binding, and assembly and remodeling of the ECM (Figure S1B; Data S2, Supporting Information). Furthermore, ECM‐associated genes (the matrisome)^[^ ^24^ ^]^ accounted for 9.7% of the upregulated genes and 2.9% of the downregulated genes (Figure S1C,D). This suggested that the brain ECM plays a significant role in the etiology of AD.^[^ ^2^ ^]^

Analysis of the proteomic data for the mPFC revealed that ChABC administration in AD mice markedly changed the expression of ECM components and ECM‐associated proteins (Figure 1B; Figure S1F,G, Supporting Information). Furthermore, gene ontology (GO) enrichment analysis indicated that the differentially expressed proteins identified in the mPFC were mainly associated with extracellular matrix structural constituent conferring tensile strength and compression resistance, hyaluronic acid binding, and extracellular matrix structural constituent (Figure 1C). Furthermore, RNA sequencing results showed that 53 genes differentially expressed in the mPFC after ChABC treatment were associated with the ECM^[^ ^24^ ^]^ (Figure S2A, Supporting Information, blue dots). Of these, 14 coded for proteins classified as ECM regulators, and 8 encoded ECM‐associated proteins that were significantly upregulated after ChABC treatment (Figure S2B, Supporting Information). These included several ECM‐degrading enzymes (ADAM5, ADAMTS1, ADAMTS9, ADAMTS14, MMP3, and MMP25) (Figure S2C,D, Supporting Information). Interestingly, we identified a strong activation signature for ECM remodeling proteins after ChABC treatment, including C‐type lectin domain family members, a matrix metallopeptidase, and multiple chemokines (Figure S2, Supporting Information). Among the top upregulated genes were *Cxcl13*, encoding a protein that binds to extracellular matrix components,^[^ ^25^ ^]^ as well as C‐type lectin domain family 4 member A (*Clec4a*) and *Adamts1*, which code for extracellular matrix remodeling factors.^[^ ^26^, ^27^ ^]^ The ECM is continuously undergoing remodeling in accordance with the brain's environment. However, in the case of AD, this remodeling process is impeded, whereas ChABC treatment facilitates and enhances the remodeling process.

ECM components are synthesized and secreted by the cell into extracellular compositions that wrap around the cell, which, on the one hand, provides a microenvironment for the cell and, on the other hand, restricts the exchange of information between cells. To exclude the interference of intracellular components, the ECM was decellularized, after which changes in ECM components were analyzed (Figure 1D). Our results showed that the expression of fibronectin and versican was slightly decreased in 5xFAD mice treated with denatured ChABC (5xFAD‐denatured ChABC mice) compared with that in WT mice treated in the same manner (WT‐denatured ChABC mice); however, the expression of both components was significantly higher in 5xFAD mice treated with ChABC (5xFAD‐ChABC mice) than in 5xFAD‐denatured ChABC mice (Figure 1E,F; Figure S3, Supporting Information). In contrast, the expression of aggrecan (ACAN) was significantly decreased in 5xFAD mice under ChABC treatment compared with that under denatured ChABC treatment (Figure 1G). ACAN is a core component of the central nervous system ECM and plays a role in synaptic plasticity and learning and memory processes.^[^ ^28^ ^]^ Interestingly, we found that ACAN was abundantly present in the core of Aβ plaques (Figure S3A, Supporting Information). An immunofluorescence assay revealed that the recruitment of astrocytes around Aβ plaques and the co‐localization of ACAN with Aβ plaques were decreased under ECM‐remodeling conditions (Figure S3A, Supporting Information); however, no significant changes were observed in the co‐localization of versican and fibronectin with Aβ plaques (Figure S3B,C, Supporting Information). The above data collectively suggested that ChABC facilitates ECM remodeling in the mPFC of AD model mice.

### 2.2 ECM Remodeling Reversed Short‐Term Spatial Memory Defects in Mice with Early‐Onset AD

**Figure 2:**
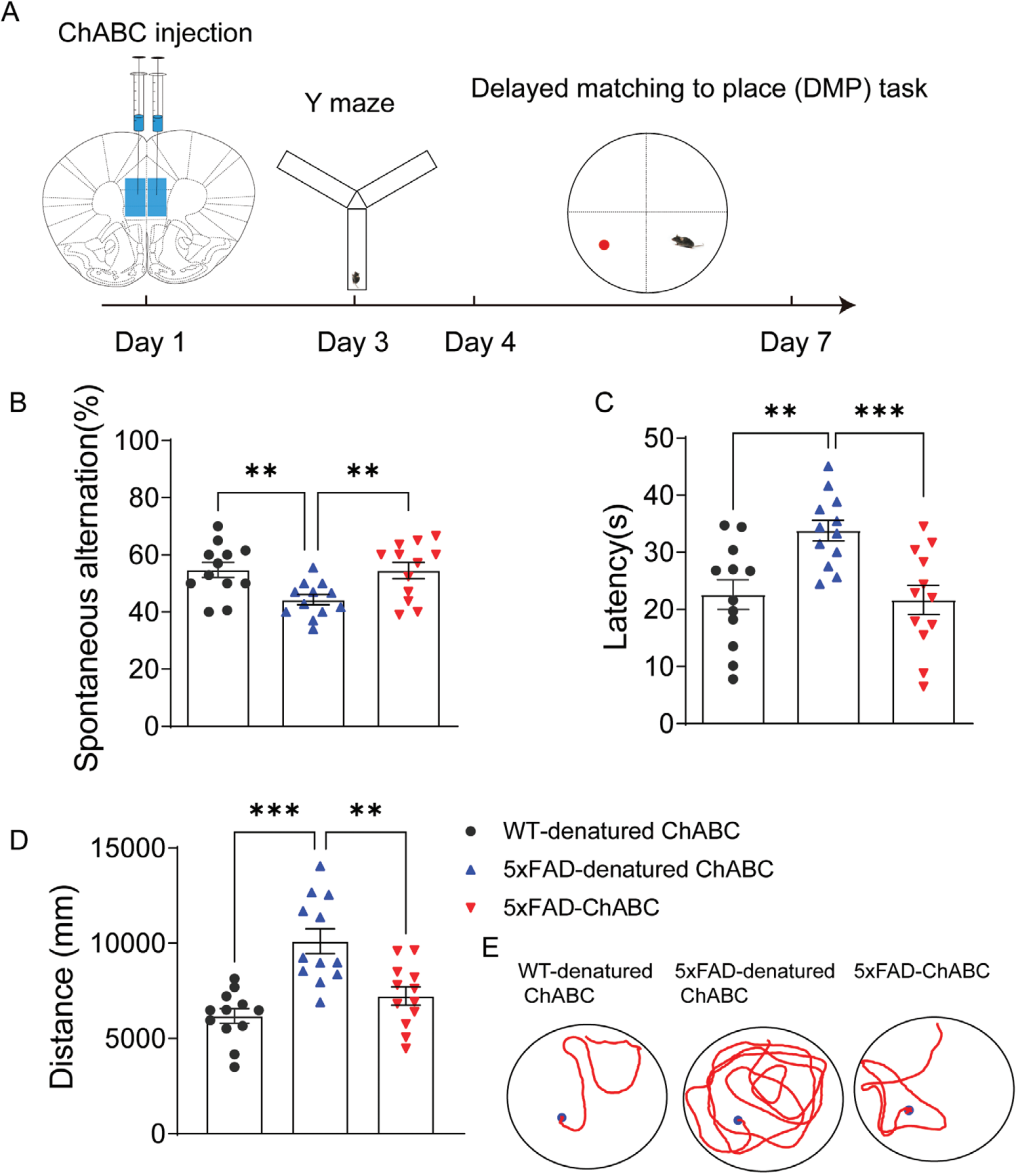
Extracellular matrix remodeling reverses the short‐term spatial memory defects in early‐onset AD model mice. A) The schematic illustration of the ChABC injection, the Y maze, and delayed matching to place task. The ChABC was injected on day 1, the Y maze was performed on day 3, and DMP was performed from day 4 to day 7. B) The percentage of spontaneous alternation among WT‐denatured ChABC mice, 5xFAD‐denatured ChABC mice, and 5xFAD‐ChABC mice. C) The escape latency among the three groups. D) The swimming distance among the three groups. E) The swimming pathway for the three groups. Data in (B), (C), and (D) are means ± SEM (numbers in bars show biological replicates). Statistical analyses were performed with One‐way ANOVA with Bonferroni's multiple comparisons test, **P < 0.01, ***P < 0.001. For additional data, see Figure S4 and S6 (Supporting Information).

Next, we investigated whether ECM remodeling in the mPFC can alleviate short‐term spatial memory impairment in AD model mice. For this, ChABC was delivered into the mPFC of 5‐month‐old AD mice, and behavioral tests to assess spatial short‐term memory were conducted after 3 days (**Figure** **2**A). In the Y‐maze test, the percentage of spontaneous alternation was markedly lower in 5xFAD‐denatured ChABC mice than in WT‐denatured ChABC animals but was significantly higher in mice of the 5xFAD‐ChABC group than in animals of the 5xFAD‐denatured ChABC group (Figure 2B). Additionally, the total number of entries into the arms of the maze was similar among the three groups (Figure S4A, Supporting Information). We also used the delayed matching to place (DMP) task to assess the restoration of spatial short‐term memory in AD mice. The results showed that the escape latency was significantly increased in 5xFAD mice compared with that in 5xFAD‐ChABC mice (Figure 2C). Similarly, the escape distance was longer among 5xFAD‐denatured ChABC mice than among WT‐denatured ChABC mice but was significantly lower in the 5xFAD‐ChABC group than in the 5xFAD‐denatured ChABC group (Figure 2D,E). No difference in swimming speed was detected among these groups (Figure S4B, Supporting Information). We further examined the spatial long‐term memory of these mice using the Morris water maze test (Figure S5, Supporting Information). We observed that, compared with their WT counterparts, the escape latency was markedly increased in 5xFAD‐ChABC and 5xFAD‐denatured ChABC mice; however, there was no difference in escape latency between the two latter groups (Figure S5B, Supporting Information). The number of platform location crossings and the percentage of time spent in the target quadrant were significantly decreased in 5xFAD‐ChABC and 5xFAD‐denatured ChABC mice compared with that in their WT counterparts (Figure S5C,D, Supporting Information). The swimming velocity did not differ among the four groups (Figure S5E, Supporting Information). These data revealed that the administration of ChABC into the mPFC did not affect long‐term memory in AD mice. Collectively, our findings suggested that applying ChABC to the mPFC can restore spatial short‐term memory in AD model mice.

To clarify whether ChABC can improve cognitive behavior in older AD mice, we injected ChABC into the mPFC of 9‐month‐old 5xFAD mice, and assessed the effect on spatial learning and memory behaviors (Figure S6, Supporting Information). The results of the Y‐maze test showed that the percentage of spontaneous alternation was notably reduced in 5xFAD‐denatured ChABC mice compared with that in WT‐denatured ChABC animals; however, there was no difference in this parameter between the 5xFAD‐ChABC and 5xFAD‐denatured ChABC treatment groups (Figure S6B, Supporting Information). Moreover, the escape latency was longer in 5xFAD‐denatured ChABC mice than in WT‐denatured ChABC mice but did not differ between 5xFAD‐ChABC and 5xFAD‐denatured ChABC mice (Figure S6C, Supporting Information). Additionally, no differences in spontaneous alternation and escape latency were detected between WT‐denatured ChABC mice and WT‐ChABC mice, suggesting that ChABC administration in the mPFC did not affect the memory ability of WT mice (Figure S4, Supporting Information). These results indicated that ECM remodeling in the mPFC did not affect spatial memory in middle or late AD. Collectively, our observations implied that ECM remodeling reverses spatial short‐term memory defects in mice with early‐onset AD.

### 2.3 ECM Remodeling Alleviated Aβ Pathology and Promoted Astrocyte Activation in the mPFC

**Figure 3:**
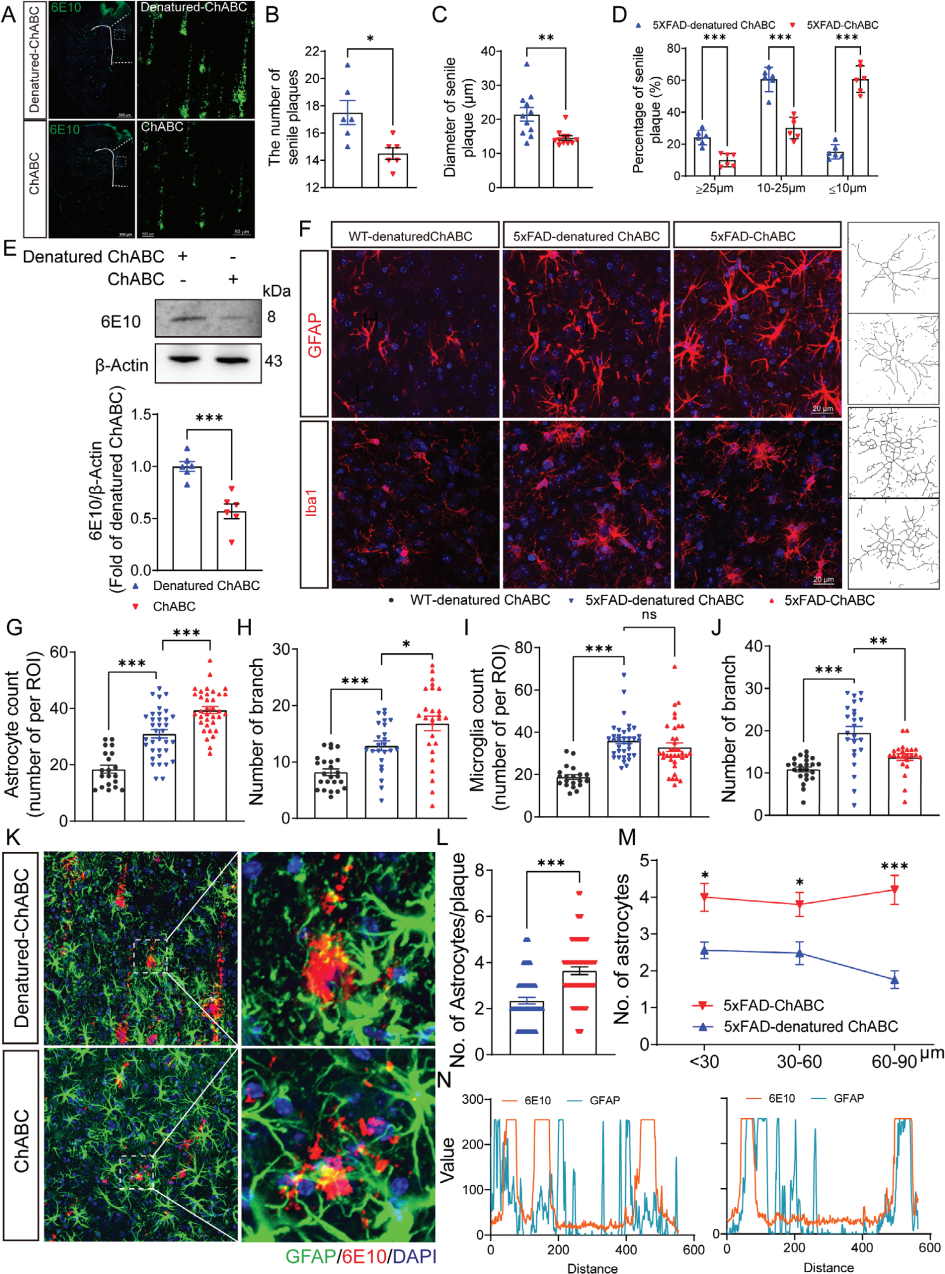
ECM remodeling alleviates Aβ pathology and reactivates astrocytes in the mPFC. A) Represent image of 6E10 in mPFC in the denatured ChABC and ChABC groups. The scale bar is 500 µm or 50 µm. B,C) Quantification of the number (B) and diameter (C) of senile plaques in the denatured ChABC and ChABC groups. D) Quantification of the percentage of senile plaques that are greater than or equal to 25 µm, between 10 and 25 µm, and less than or equal to 10 µm in the denatured ChABC and ChABC groups. E) Western blotting analysis of 6E10 in PFC of 5xFAD mice after ChABC or denatured ChABC treatment. F) Immunofluorescent images of GFAP and Iba1 in the mPFC from WT‐denatured ChABC, 5xFAD‐denatured ChABC, and 5xFAD‐ChABC mice. The scale bar is 20 µm. G,H) Quantification of the astrocyte cell number (G) and the number of branch (H) in the mPFC from WT‐ denatured ChABC, 5xFAD‐denatured ChABC, and 5xFAD‐ChABC mice. I,J) Quantification of the microglial cell number (I) and the number of branch (J) in the mPFC from WT‐ denatured ChABC, 5xFAD‐denatured ChABC, and 5xFAD‐ChABC mice. K) Immunofluorescent images of 6E10 (red) and GFAP (green) in mPFC from 5xFAD mice after denatured ChABC or ChABC administration. The scale bar is 20 µm. L) Quantification of the number of astrocytes per plaque in the 5xFAD‐denatured ChABC and 5xFAD‐ChABC mice. M) Quantification of the number of astrocytes from 30 µm, 30 to 60 µm, and 60 to 90 µm around Aβ plaques in the 5xFAD‐denatured ChABC and 5xFAD‐ ChABC mice. N) Colocalization analysis of 6E10 and GFAP from 5xFAD‐denatured ChABC mice (left) and 5xFAD‐ChABC mice (right). Data in (B) – (E), (G) – (J), (L), and (M) are means ± SEM (numbers in bars show cells/ biological replicates). Statistical analyses were performed by unpaired two‐sided Student's t‐test in (B), (C), (E), and (L), Two‐way ANOVA with Bonferroni's multiple comparisons test in (D) and (M), or Brown‐Forsythe and Welch ANOVA with Bonferroni's multiple comparisons test in (G) to (J), *P < 0.05, **P < 0.01, ***P < 0.001, ns, no significant. For additional data, see Figure S7 and S8.

Next, we sought to elucidate whether ECM remodeling can alleviate Aβ pathology in the mPFC, a key hallmark of AD. Sections of the mPFC were immunostained with the 6E10 antibody or stained with thioflavin‐S, and the number and diameter of Aβ plaques were quantified. Compared with 5xFAD‐denatured ChABC mice, 5xFAD mice administered ChABC exhibited a noticeable reduction in Aβ deposition (**Figure** **3**A–C; Figure S7A, Supporting Information). Additionally, to further clarify the effect of ChABC on Aβ plaques, plaque sizes were compared among the groups. Compared with 5xFAD‐denatured ChABC mice, the number of plaques with a diameter greater than 25 µm or between 10 and 25 µm was greatly decreased in 5xFAD‐ChABC mice, whereas the number of plaques smaller than 10 µm in diameter was significantly increased (Figure 3D). These results indicated that ECM remodeling reduces Aβ deposition. To further assess whether ECM remodeling affects Aβ protein production, the expression levels of amyloid precursor protein (APP), the C‐terminal APP fragment, and β‐site APP cleaving enzyme 1 (BACE1) were measured by western blot (Figure S7B, Supporting Information). Compared with WT mice, the levels of these proteins were noticeably increased in 5xFAD mice; however, the levels of these proteins did not differ between 5xFAD‐denatured ChABC and 5xFAD‐ChABC mice (Figure S7C–E, Supporting Information). Meanwhile, we found that ECM remodeling did not affect the expression of Aβ‐degrading enzymes,^[^ ^29^, ^30^ ^]^ including tissue‐type plasminogen activator (*tPA*), insulin‐degrading enzyme (*IDE*), neprilysin (*NEP*), and angiotensin‐converting enzyme (*ACE*) in the mPFC (Figure S7F, Supporting Information). In addition, the Aβ level was significantly decreased in 5xFAD mice following ChABC treatment (Figure 3E). Our findings suggested that ECM remodeling reduces Aβ deposition without affecting Aβ production.

Astrocyte and microglia activation are important hallmarks of AD pathology. To determine whether glial cells are activated following the injection of ChABC into the mPFC in AD mice, astrocytes and microglia were immunostained with anti‐GFAP and anti‐Iba1 antibodies. The results showed that both astrocytes and microglia were activated in AD mice, as evidenced by the observed increase in the number, cell body area, and branch complexity of astrocytes and microglia in 5xFAD‐denatured ChABC mice relative to that in WT‐denatured ChABC mice (Figure 3F–J; Figure S7, Supporting Information). In addition, the protrusion length of astrocytes was increased and that of microglia was decreased (Figure S7, Supporting Information), which was consistent with previous studies.^[^ ^31^ ^]^ The number of astrocytes and astrocyte branching showed a marked increase in 5xFAD‐ChABC mice (Figure 3G,H); however, ChABC treatment did not affect the cell body area or the protrusion length of astrocytes (Figure S7, Supporting Information). Furthermore, the number and cell body area of microglia did not differ between the 5xFAD‐ChABC group 5xFAD‐denatured ChABC mice (Figure 3I; Figure S7, Supporting Information), although the protrusion length was slightly increased (Figure S7, Supporting Information) and the branch number was slightly decreased (Figure 3J). These results indicated that ECM remodeling promoted the activation of astrocytes, but not microglia, in the mPFC of AD model mice. Together, these findings demonstrated that ECM remodeling alleviates Aβ pathology and activates astrocytes in the mPFC.

### 2.4 ECM Remodeling Promoted Astrocyte Recruitment around Plaques and Aβ Phagocytosis

An imbalance between Aβ production and clearance leads to Aβ accumulation and aggregation in the brain.^[^ ^32^, ^33^ ^]^ Here, we found that ECM remodeling resulted in a reduction in Aβ deposition without a concomitant decrease in Aβ production in AD mice; accordingly, we next sought to determine whether ChABC treatment contributed to Aβ clearance. Microglia and astrocytes are proposed to take up and clear Aβ plaques.^[^ ^33^, ^34^ ^]^ Our immunofluorescence data showed that astrocytes and microglia clustered around Aβ plaques in 5xFAD mice (Figure 3K; Figure S8A, Supporting Information). ChABC treatment significantly enhanced the recruitment of astrocytes, but not microglia, to Aβ plaques (Figure 3K,L; Figure S8A, Supporting Information). Additionally, ChABC treatment resulted in increased astrocyte clustering around Aβ plaques in 5xFAD mice (Figure 3M). These results suggested that ECM remodeling promotes astrocyte recruitment around Aβ plaques.

To elucidate whether the enhanced astrocyte recruitment around plaques facilitated Aβ engulfment and clearance, we analyzed the colocalization of astrocytes with Aβ plaques. The results showed that the colocalization of astrocytes, but not microglia, with Aβ plaques were significantly enhanced with ChABC treatment (Figure 3N; Figure S8B, Supporting Information). Together, these findings suggested that ChABC treatment enhances Aβ engulfment and clearance by astrocytes.

To further demonstrate that ChABC can enhance astrocyte‐mediated Aβ phagocytosis, astrocytes were isolated from mouse brains for primary culture and then subjected to Aβ_1–42_‐FITC uptake assays (Figure S8C, Supporting Information). Consistent with the colocalization analysis, the engulfment of Aβ_1–42_‐FITC oligomers and polymers by astrocytes was significantly increased following ChABC treatment (Figure S8 E and E, Supporting Information). However, ChABC administration did not influence Aβ_1–42_‐FITC oligomer phagocytosis, and slightly decreased Aβ_1–42_‐FITC polymer phagocytosis by microglia (Figure S8F,G, Supporting Information). Together, these results indicated that ChABC stimulates the recruitment of astrocytes adjacent to Aβ plaques and improves their phagocytic capacity, thereby promoting Aβ clearance in AD mice.

### 2.5 ECM Remodeling Alleviated AD Pathology by Enhancing Autophagic Flux

**Figure 4:**
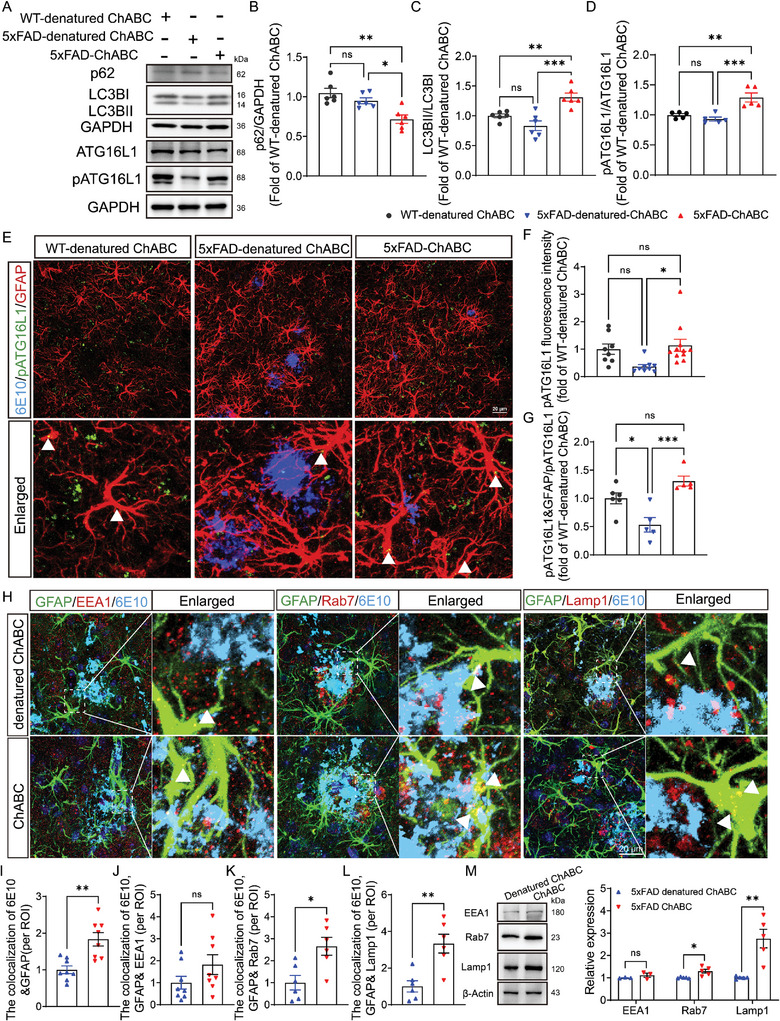
ECM remodeling enhances the astrocytic autolysosome pathway and Aβ clearance. A) Western blotting analysis of p62, LC3B I/II, ATG16L1, and pATG16L1 in PFC from WT‐denatured ChABC, 5xFAD‐denatured ChABC, and 5xFAD‐ChABC mice. B–D) Quantification of the expression level of p62 (B), LC3BII/LCB3I (C), and pATG16L1/ATG16L1 (D) in the western blotting shown in (A). E) Immunofluorescent images of 6E10 (blue), pATG16L1 (green), and GFAP (red) in mPFC from 5xFAD mice and WT mice after denatured ChABC or ChABC administration. The scale bar is 20 µm. F) Quantification of the fluorescence intensity of pATG16L1 in the WT‐denatured ChABC, 5xFAD‐denatured ChABC, and 5xFAD ChABC mice. G) Quantification of the fold of pATG16L1 per astrocytes to total pATG16L1 in the WT‐denatured ChABC, 5xFAD‐denatured ChABC, and 5xFAD‐ ChABC mice. H) Immunofluorescent images of GFAP (green), 6E10 (cyan), EEA1 (red), Rab7 (red), and Lamp1 (red) in mPFC from 5xFAD mice after denatured ChABC or ChABC administration. The scale bar is 20 µm. I–L) Quantification of the colocalization of 6E10 and GFAP (I), 6E10, GFAP and EEA1 (J), 6E10, GFAP, and Rab7(K), and 6E10, GFAP and Lamp1(L) from the 5xFAD mice after ECM remodeling. M) Western blotting analysis of EEA1, Rab7, and Lamp1 from the 5xFAD mice after ECM remodeling. Data in (B) – (D), (F) – (G), and (I) – (M) are means ± SEM (numbers in bars show biological replicates). Statistical analyses were performed by One‐way ANOVA with Bonferroni's multiple comparisons test in (B) to (D) and (F) to (G), unpaired two‐sided Student's t‐test in (I) to (L), or Two‐way ANOVA with Bonferroni's multiple comparisons test in (M), *P < 0.05, **P < 0.01, ***P < 0.001, ns, no significant. For additional data, see Figure S9 and S10 (Supporting Information).

ECM proteins regulate autophagy flux bidirectionally, and autophagy subsequently affects ECM remodeling.^[^ ^21^, ^22^, ^35^ ^]^ To determine how ECM remodeling alleviates AD pathology, we examined autophagic flux under ECM remodeling conditions. The expression of classic markers of autophagy, including p62 and LC3II, and that of ATG16L1(Ser278) (pATG16L1), a recently identified marker of autophagy,^[^ ^36^ ^]^ was analyzed by western blot (**Figure** **4**A). We found that the expression of p62 as well as the pATG16L1/ATG16L1 ratio and LC3BII/LC3BI ratio, in the 5xFAD‐denatured ChABC group, were comparable to those in the WT‐denatured ChABC group (Figure 4B–D). However, compared with the 5xFAD‐denatured ChABC group, the expression of p62 was significantly decreased in the 5xFAD‐ChABC group (Figure 4B, *P* = 0.02), whereas the LC3BII/LC3BI and pATG16L1/ATG16L1 ratios were substantially higher (Figure 4C,D, *P* < 0.001). These findings indicated that autophagic flux was enhanced under ECM remodeling in AD mice.

Next, pATG16L1, 6E10, and GFAP fluorescence staining were used to examine the relationship between alterations in autophagic flux and AD pathology in astrocytes during ECM remodeling. We found that the number of pATG16L1‐positive puncta was noticeably decreased in the mPFC of 5xFAD‐denatured ChABC mice compared with that in WT‐denatured ChABC mice. The number of pATG16L1‐positive puncta in 5xFAD‐ChABC mice was significantly higher than that in 5xFAD‐denatured ChABC mice and was comparable to that in WT‐denatured ChABC mice (Figure 4E). Quantification of the pATG16L1 fluorescence intensity also demonstrated that the pATG16L1 levels were significantly increased in 5xFAD mice treated with ChABC relative to that in 5xFAD mice treated with denatured ChABC (Figure 4F, *P* = 0.007). Additionally, the percentage of pATG16L1 puncta in astrocytes was slightly lower in 5xFAD‐denatured ChABC mice than in WT‐denatured ChABC mice (*P* = 0.02), but was significantly higher in 5xFAD‐ChABC mice than in 5xFAD‐denatured ChABC mice (Figure 4G, *P*<0.001). Similarly, transmission electron microscopic (TEM) analysis showed that the number of autophagosomes was significantly increased under ECM remodeling (Figure S9A,B, Supporting Information). To confirm the effect of ChABC on autophagy flux, we utilized a double‐tagged LC3B (eGFP‐mCherry‐LC3B) found that the number of autophagosomes and autolysosomes increased in ChABC‐induced cells that compared with control cells (Figure S9C,D, Supporting Information). Together, our data revealed that ECM remodeling in AD mice enhances astrocyte‐mediated autophagic flux.

### 2.6 ECM Remodeling Activated the Astrocytic Lysosomal Pathway, Leading to Aβ Degradation

Mounting evidence suggests that ECM remodeling can modulate the intracellular autophagy‐lysosome pathway and thus regulate cellular degeneration.^[^ ^21^, ^37^, ^38^ ^]^ Here, we postulated that ECM remodeling triggers the activation of the astrocytic lysosomal pathway, thereby augmenting phagocytic activity in astrocytes and facilitating the clearance of Aβ. To test this, we investigated the colocalization of GFAP and 6E10 with EEA1, an early endosomal marker; Rab7, a late endosomal marker; or LAMP1, a lysosomal marker, using an immunofluorescence assay (Figure 4H). We found greater numbers of 6E10‐positive puncta (Figure 4I, *P* = 0.0014) and 6E10/EEA1 double‐positive puncta (Figure 4J, *P* = 0.1483) in astrocytes of the 5xFAD‐ChABC group than in those of the 5xFAD‐denatured ChABC group. However, no difference in the number of 6E10/EEA1 double‐positive (GFAP‐negative) puncta was observed between the two groups (Figure S10A, Supporting Information; *P* = 0.0558). Notably, colocalization among GFAP, 6E10, and Rab7 (Figure 4K, *P* = 0.0071), as well as that between 6E10 and Rab7 (Figure S10B, Supporting Information; *P* = 0.0101), showed a noticeable increase in the ChABC group. Furthermore, colocalization among GFAP, 6E10, and Lamp1 (Figure 4L, *P* = 0.0286), as well as that between 6E10 and Lamp1 (Figure S10C, Supporting Information *P* = 0.0028), demonstrated a significant increase in the ChABC group compared with that in the denatured ChABC group. Consistent with the immunofluorescence assay, western blotting results indicated that the expression level of EEA1 was unaffected under ChABC treatment, while those of Rab7 and Lamp1 were significantly increased under the same condition (Figure 4M). Furthermore, treatment with the lysosomal inhibitor bafilomycin A (BafA1) markedly reduced the intensity of FITC‐Aβ_1–42_ puncta in primary astrocytes. Nevertheless, the intensity of FITC‐Aβ_1–42_ puncta did not differ between the BafA1 and ChABC co‐treatment group and the group treated only with BafA1 (Figure S10D, Supporting Information), indicating that the lysosomal pathway is the primary route by which ECM remodeling promotes Aβ elimination by astrocytes. Combined, these findings suggested that ECM remodeling may facilitate astrocyte‐mediated internalization and elimination of Aβ plaques by boosting the lysosomal pathway.

### 2.7 ECM Remodeling Enhanced Phagocytosis by Astrocytes

**Figure 5:**
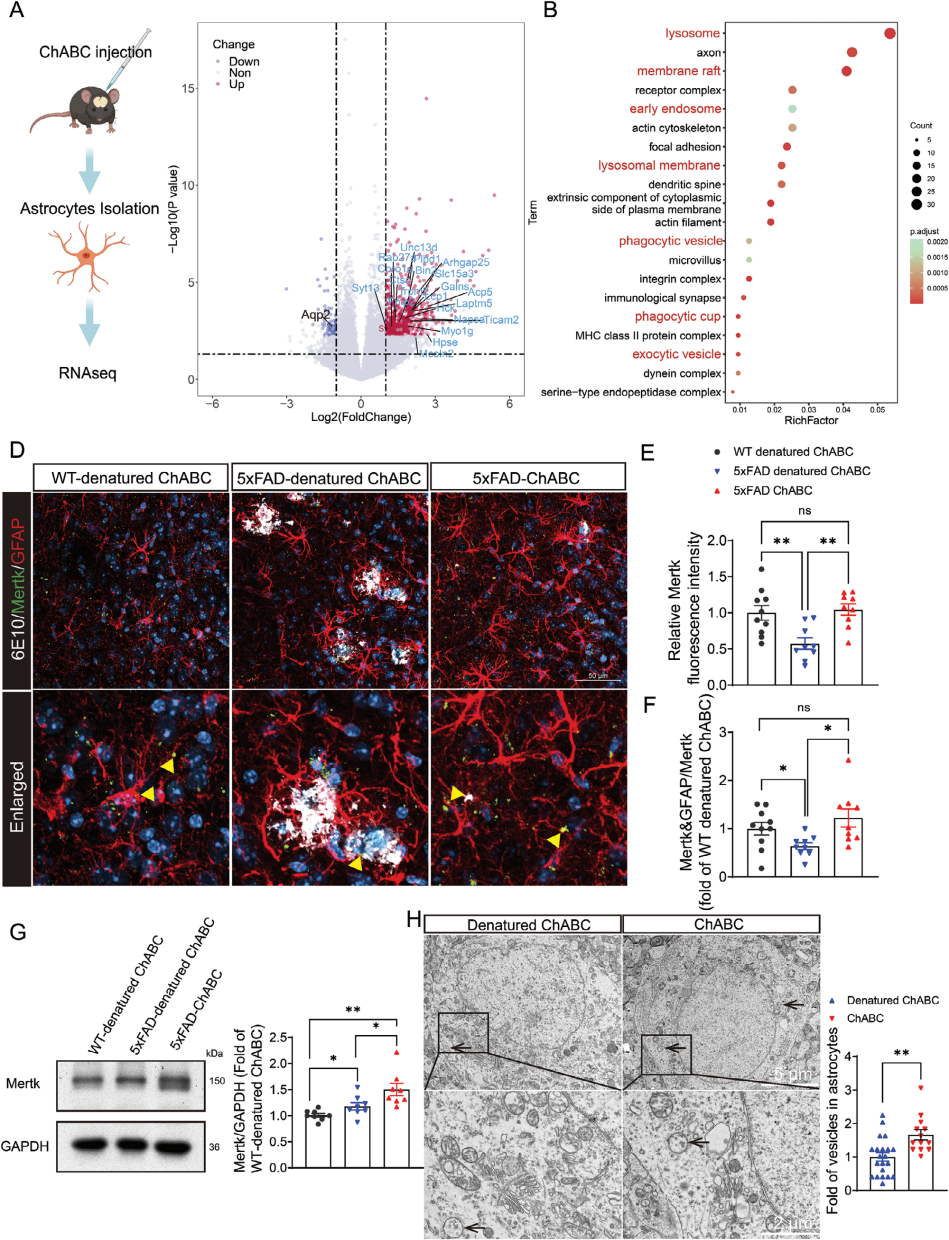
ECM remodeling activates the expressions of phagocytic receptors and increases vesicles in astrocytes. A) Astrocytes were isolated into PFC from WT‐denatured ChABC, 5xFAD‐denatured ChABC and 5xFAD‐ChABC mice, then RNAseq was performed (PFC from 5–6 mice mixed into one sequencing sample). B) The volcano plot showed the upregulation and downregulation of genes in mPFC astrocytes of 5xFAD mice after administration of ChABC and denatured ChABC. C) Bubble plot of significantly enriched top 20 Gene Ontology (GO) terms of differentially expressed genes screened by RNAseq in PFC astrocytes from 5xFAD mice after ChABC and denatured ChABC administration. D) Immunofluorescent images of Mertk(green), GFAP (red) and 6E10 (white) in mPFC from 5xFAD mice and WT mice after denatured ChABC or ChABC administration. The scale bar is 50 µm. E) Quantification of the fluorescence intensity of Mertk in the WT‐denatured ChABC, 5xFAD‐denatured ChABC, and 5xFAD‐ChABC mice. F) Quantification of the fold of Mertk per astrocytes to total Mertk (white triangle) in the WT‐denatured ChABC, 5xFAD‐denatured ChABC, and 5xFAD‐ChABC mice. G) Western blot analysis of Mertk in PFC from WT‐denatured ChABC, 5xFAD‐denatured ChABC, and 5xFAD‐ChABC mice (*n* = 3). Quantification of the expression level of Mertk in the western blotting. H) Transmission electron microscope images of the vesicles (black arrow) in astrocytes in PFC from 5xFAD mice after denatured ChABC or ChABC administration. The right panel is the quantification of the vesicles in astrocyte. The top image scale bar is 5 µm, and the down image scale bar is 2 µm. Data in (E) to (H) are means ± SEM (numbers in bars show biological replicates/ cells). Statistical analyses were performed by One‐way ANOVA with Bonferroni's multiple comparisons test in (E) to (G), unpaired two‐sided Student's *t*‐test in (H), *P < 0.05, **P < 0.01, ns, no significant. For additional data, see Figure S11 and S12 (Supporting Information).

To clarify the mechanism by which astrocytes are recruited around plaques for the purpose of phagocytosing Aβ, astrocytes were isolated from the mPFC after ECM remodeling, and subjected to transcriptome sequencing (**Figure** **5**A). The results revealed that the expression of genes associated with lysosomes, phagocytosis, and vesicles was markedly upregulated following ChABC administration (Figure 5B,C; Figure S11, Supporting Information). Astrocytes express various phagocytic receptors, such as MEGF10 and MERTK, that are involved in synapse and Aβ plaque elimination.^[^ ^39^, ^40^, ^41^ ^]^ Consequently, we wondered whether ECM remodeling enhances astrocyte‐mediated phagocytosis of Aβ plaques by activating phagocytic receptors on astrocytes. Immunofluorescence results revealed that the fluorescence intensity of MERTK was significantly reduced in the 5xFAD‐denatured ChABC group when compared with that in both the WT‐denatured ChABC and 5xFAD‐ChABC groups (Figure 5D,E). Similarly, the MERTK signal in astrocytes was slightly decreased in the 5xFAD‐denatured ChABC group compared with that in both the WT‐denatured ChABC group and the 5xFAD‐ChABC group (Figure 5F). Furthermore, the protein level of MERTK was slightly higher in the 5xFAD‐denatured ChABC group than in the WT‐denatured ChABC group but was significantly higher in the 5xFAD‐ChABC group than in the other two groups (Figure 5G). Meanwhile, the protein level of MEGF10 did not differ among the three groups (Figure S12A, Supporting Information). These results revealed that ChABC activates phagocytic receptor MERTK on astrocytes to engulf Aβ plaques.

Vesicles serve as the primary means for intracellular cargo transportation and recycling. To further confirm that ECM remodeling can increase vesicle numbers, TEM images were collected 7 days after the delivery of ChABC to the mPFC of 5xFAD mice. The results showed that vesicle numbers were significantly increased in astrocytes (Figure 5H), but not microglia or neurons (Figure S12B,C, Supporting Information). Collectively, these data suggested that ECM remodeling promotes astrocyte‐mediated Aβ phagocytosis by activating astrocyte phagocytosis and increasing the number of astrocytic vesicles.

## 3 Discussion

The ECM plays a crucial role in maintaining a homeostatic microenvironment in the brain. However, during the pathological process of AD, various components of the ECM change and contribute to the pathology of the disease.^[^ ^2^, ^7^, ^10^ ^]^ Nevertheless, the specific impact of changes in ECM composition, also known as ECM remodeling, on the progression of AD remains unclear. In this study, we demonstrated that ECM remodeling leads to the restoration of short‐term memory loss and a reduction in Aβ deposition in the mPFC of early‐onset 5xFAD model mice. Furthermore, we revealed that ECM remodeling activates the astrocyte phagocytic receptor and, subsequently, the autophagy‐lysosomal pathway, thereby enhancing vesicle recycling for phagocytosis and the removal of accumulated Aβ. Our work has uncovered a cellular mechanism by which ECM remodeling mitigates AD pathology by activating the astrocytic autophagy‐lysosomal pathway.

The brain ECM is a highly dynamic structural network with regional variation that continuously undergoes remodeling mediated by several matrix‐degrading enzymes during both normal and pathological conditions.^[^ ^4^, ^42^, ^43^ ^]^ In addition to its crucial role in supporting cellular morphology and structure, the ECM is also intricately involved in processes such as cell survival, growth, migration, and differentiation, and makes a vital contribution to the maintenance of cellular and tissue homeostasis. Our data showed that some ECM regulators and ECM‐associated proteins are significantly upregulated after the intracerebral injection of ChABC into the mPFC of 5‐month‐old 5xFAD mice. Additionally, the expression of matrix‐degrading enzymes, including ADAM5, ADAMTS1, ADAMTS9, ADAMTS14, MMP3, and MMP25, which are extracellular matrix remodeling factors,^[^ ^4^, ^26^, ^27^ ^]^ were markedly upregulated in the 5xFAD‐ChABC group. ECM components are found in Aβ plaques. Aβ, and, in particular, its aggregated forms, can bind a range of ECM‐associated proteins.^[^ ^7^, ^44^ ^]^ Studies have shown that the levels of ECM components exhibit spatiotemporal specificity in the AD brain, and alterations in the ECM occur during the early stages of AD.^[^ ^7^, ^8^, ^45^ ^]^ Our proteomic and decellularization results also showed changes in ECM proteins and suggested that ChABC facilitates ECM remodeling.

ECM proteins and proteoglycans have been found in Aβ plaques, and have been reported to modulate Aβ aggregation and deposition.^[^ ^7^ ^]^ The matrisome (the collection of ECM‐associated proteins) co‐expression module shows a strong correlation with AD endophenotypes.^[^ ^46^ ^]^ Some glycosaminoglycans and proteoglycans can enhance the deposition of beta2‐microglobulin‐related (beta2m) amyloid fibrils in vivo, possibly by binding directly to the surface of the fibrils and stabilizing the conformation of beta2m in the fibrils, as well as by acting as a scaffold for the polymerization of beta2m into the fibrils.^[^ ^47^ ^]^ The intracerebral injection of ChABC into the hippocampus of APPswe/PS1dE9 mice has been shown to disrupt the CSPG network, resulting in a reduction in Aβ plaque numbers, enhanced synaptic plasticity, and the restoration of contextual memory performance.^[^ ^23^, ^48^ ^]^ We found that the intracerebral injection of ChABC into the mPFC of 5‐month‐old 5xFAD mice effectively restored spatial short‐term memory but not long‐term memory in the animals. This finding aligns with existing literature highlighting the distinct mechanisms and brain regions involved in short‐term versus long‐term memory processing.^[^ ^49^ ^]^ Specifically, the mPFC is predominantly associated with short‐term memory functions, such as working memory,^[^ ^50^, ^51^ ^]^ whereas long‐term memory formation is primarily attributed to the hippocampus.^[^ ^52^, ^53^ ^]^ Our results are consistent with previous studies demonstrating that ECM remodeling enhances neuroplasticity and short‐term memory.^[^ ^54^, ^55^ ^]^ Furthermore, our study revealed that ChABC treatment did not lead to an improvement in spatial memory among 9‐month‐old AD mice. This may be attributed to the stiffness of the ECM in the brains of these mice,^[^ ^56^, ^57^ ^]^ which makes remodeling challenging, resulting in levels of ECM remodeling that may not be adequate for inducing behavioral changes. The combined use of multiple extracellular matrix hydrolases such as matrix metalloproteinase, heparinase, could be a promising strategy to remodel the stiffness of ECM of 9‐month‐aged AD mice. These observations suggest that ChABC promotes ECM remodeling, which, in turn, improves AD‐related short‐term memory behaviors.

Our data also showed that ECM remodeling leads to a reduction in the size of Aβ plaques without simultaneously influencing Aβ production, suggesting that altered ECM components may interact with Aβ plaques directly, a possibility that needs further investigation. Astrocyte and microglia activation are prominent features of AD pathology, and the early activation of these glial cells promotes Aβ clearance.^[^ ^33^, ^58^ ^]^ Here, we found that ECM remodeling selectively activates astrocytes, but not microglia, and recruits them to the sites of Aβ plaques for plaque clearance. In vitro experiments further confirmed that ChABC promotes Aβ phagocytosis by astrocytes rather than microglia. These data suggest that ECM remodeling alleviates cognitive impairment by acting on astrocyte phagocytosis and clearing Aβ deposition.

The autophagy‐lysosomal pathway represents a means by which cellular material is degraded by lysosomes or vacuoles and is subsequently recycled, and is also a means via which cells continuously eliminate toxic components. Interestingly, studies have demonstrated that ECM remodeling regulates autophagic activity bidirectionally.^[^ ^21^, ^22^, ^35^, ^37^, ^38^ ^]^ The expression level of pATG16L1 represents a newly tool to monitor autophagy induction.^[^ ^36^, ^59^ ^]^ In this study, we suggest that, under ECM remodeling, the levels of autophagy were noticeably enhanced in the mPFC of 5xFAD mice, especially in astrocytes. Accumulating evidence has indicated that impaired autophagy contributes to AD pathogenesis. The core subunits of the VPS34 complex, Beclin‐1, and PI3P were downregulated in AD patients’ brains.^[^ ^60^, ^61^ ^]^ Herein, we did not detect alterations in autophagy levels in the PFC of AD mouse models, potentially due to regional specificity within the brain.^[^ ^62^ ^]^ ECM remodeling upregulated the expression of Rab7 and Lamp1, and increased the colocalization of Aβ with Rab7 and Lamp1 in astrocytes. In vitro experiments also suggested that Aβ phagocytosis by astrocytes is achieved through the lysosomal pathway. This implies that ECM remodeling activates the late endosome‐lysosome pathway in astrocytes for the phagocytosis of Aβ plaques. Fibronectin has been identified as a strong inducer of autophagy,^[^ ^63^ ^]^ whereas ACAN exerts the opposite effect.^[^ ^64^ ^]^ In this study, our results also showed that, under ECM remodeling conditions, the expression of fibronectin was increased, while that of ACAN was decreased, in the mPFC of 5xFAD mice. We propose that this alteration in ECM composition is the seed of astrocytic autophagy, a notion that warrants further in‐depth investigation.

Lastly, astrocyte transcriptomic data for adult mice indicated that ECM remodeling increases the expression of lysosome‐, phagocytosis‐, and vesicle‐related genes. Interestingly, we also found that the expression of the astrocyte phagocytic receptor MERTK^[^ ^40^ ^]^ and the number of intracellular vesicles both increased under ECM remodeling in AD model mice. These findings provide additional evidence that the remodeling of the ECM enhances cognitive function by stimulating the astrocytic autophagy‐lysosomal pathway, leading to the clearance of aggregated Aβ.

In summary, in this study, we elucidated the inter‐ and extracellular mechanisms underlying ECM remodeling that contribute to the restoration of spatial short‐term memory and the alleviation of Aβ pathology in AD mice. Notably, our data suggested that the activation of the astrocyte lysosomal pathway plays a significant role in these processes. These findings offer potential therapeutic strategies for AD and serve as a point of reference for the treatment of this condition.

## 4 Experimental Section

### Animals

The 5×FAD mice overexpressing the K670 N/M671L(Swedish), I716V (Florida), and V717I (London) mutations in human APP (695), as well as M146 L and L286V mutations in human PS1,^[^ ^65^ ^]^ were provided by H. Qing (Beijing Institute of Technology, Beijing, China). Mice were group‐housed in four to six on a 12/12 h light/dark cycle, and all experiments were performed during the light cycle. All mice were male and female and 5 months old. Food and water were accessed ad libitum. Littermates were randomly assigned to each condition by the experimenter. All experimental animal procedures were approved by the Institutional Animal Care and Use Committees of Yanan University (YDIACUC2020_0×051).

### Drug Treatment

Mice at 5 months old were anesthetized with sodium pentobarbital (60 mg kg^−1^) and placed in a stereotactic frame (RWD). Ophthalmic ointment was applied to prevent dehydration. 200 nL ChABC (0.04U; C3667, Sigma) or denatured ChABC were injected into bilateral mPFC (AP: 1.94 mm; ML: ±0.3 mm; DV: −2.0 mm) from the skull surface. Drug injection was performed using a 10 µL Hamilton microsyringe delivering virus at a rate of 40 nL min^−1^ with a microsyringe pump (UMP3; WPI, Sarasota, FL, USA) and controller (Micro4; WPI, Sarasota, FL, USA). After the ChABC injection was completed, the needle was held at the injection site for 5 min, then raised by 0.05 to 1 mm and held for a further 10 min to allow diffusion of the ChABC. The needle was then slowly withdrawn completely. The scalp was sutured with medical thread. Mice were allowed to recover for 3 days before behavioral experiments.

### Y‐Maze Test

The Y‐maze utilized in the experiment was comprised of three identical arms made of gray Plexiglas, each measuring 35 × 7.5 × 15 cm. Each mouse was positioned at the end of one arm and given the freedom to navigate through the maze for 5 min. The sequence of arm entries was recorded visually, and three consecutive differential choices were defined as an alternation. The percentage of alternations was calculated using the formula: (number of alternations/number of entries) × 100. Furthermore, the total number of arms entered by the mice throughout the session was also recorded.

### Delayed Matching to Place Tasks for Mice in the Morris Water Maze

The Morris water maze contained a 1.5‐meter diameter cylinder and a circular escape platform with an 8 cm diameter. The water maze procedure is similar to the previous work.^[^ ^31^ ^]^ Briefly, the position of the escape platform was altered randomly and avoided position in the same quadrant for 2 consecutive days. The experimental phases lasted four days. Four trials were performed per day with an interval of 5 min and released from N, E, S, and W sites in a different order each day. In each trial, mice could search the platform for up to 90 s. In the task, if the mouse found the platform, it could stay on the platform for 15 s before being transferred to the home cage. Otherwise, mice were picked up to the platform and kept there for 30 s. The escape latency, cumulative duration, and swimming speed were recorded using VisuTrack software (XR‐VT, Shanghai XINRUAN Information Technology Co. Ltd., China).

### Hidden Platform Tasks for Mice in Morris Water Maze

The Morris water maze with a hidden platform was used to explore long‐term memory in mice.^[^ ^66^ ^]^ Briefly, throughout the training and test days, the platform was located 1 cm under the surface of the water. On the test day, the platform was removed from the water. Throughout the experiment, mice were trained for 4 trials per day, with an intertrial interval of 20 min. The escape latency, the crossing numbers, and the swimming speed were recorded using VisuTrack software (XR‐VT, Shanghai XINRUAN Information Technology Co. Ltd., China).

### Immunofluorescence Staining

Mice were anesthetized with sodium pentobarbital (60 mg Kg^−1^) and perfused with saline. Brains were extracted and incubated in 4% paraformaldehyde for 48 h at 4°C. The brains were infiltrated in 30% sucrose (wt./vol) in PBS for at least 48 h. Coronal sections were cut at 30‐µm with a freezing microtome (1900, Leica) and stored at −20 °C in cryoprotective storage solution [300 g of sucrose, 10 g of polyvinylpyrrolidone, 500 mL of phosphate buffer (0.1 m), 300 mL of ethylene glycol, and up to 1000 mL of ddH2O] until use. Upon use, the sections were washed three times with PBS and then blocked with PBS + 0.3% Triton X‐100 (PBST) with 5% normal horse serum for 1 h at room temperature. After blocking, the sections were incubated with the primary antibody in PBST with 5% normal horse serum at 4 °C overnight. After triple washes in PBS every 5 min, sections were stained with secondary Alexa Fluor 488 AffiniPure Donkey Anti‐Mouse IgG (HL) (715‐545‐150, Jackson ImmunoResearch), Rhodamine (TRITC) AffiniPure Donkey Anti‐Goat IgG (H+L) (705‐025‐147, Jackson ImmunoResearch), and Alexa Fluor 647 AffiniPure Donkey Anti‐Rabbit IgG (H+L) (711‐605‐152, Jackson ImmunoResearch). Nuclear was stained with DAPI. Primary antibody used include goat GFAP (1:1000, Cat. No. ab53554, Abcam), rabbit Iba1 (1:1000, Cat. No. CAJ3125, Wako), mouse NeuN (1:1000, Cat. No. MAB377, Millipore), anti‐Chondroitin Sulfate antibody (named CS‐56, 1:500, Cat. No. C8035, Sigma‐Aldrich), rabbit Rab7 (1:200, Cat. No. 9367s, Cell Signaling Technology), rabbit EEA1 (1:1000, Cat. No. ab2900, Abcam), rabbit Lamp1 (1:200, Cat. No. ab24170, Abcam), mouse 6E10 (1:2000, Cat. No. 80 300, Biolegend), mouse aggrecan (1:500, Cat. No. MA3‐16888, Invitrogen), rabbit Fibronectin (1:1000, Cat. No. A12977, Abclonal), rabbit versican (1:500, Cat. No. GTX03733, GeneTex), rabbit ATG16L1(Ser278) (1:200, Cat. No. ab195242, Abcam), and rabbit MERTK (1:500, Cat. No. ab184086, Abcam). The series of fluorescent images were acquired using a Zeiss800 confocal microscope (Zeiss) or a Nikon A1R SI Confocal microscope (Nikon).

### Cell Culture and Transduction

Gl261 cells were cultured in Dulbecco's Modified Eagle's Medium (DMEM; Cat. No. C11995500BT, Gibco), supplemented with 10% fetal bovine serum (Cat. No. 04–001‐1ACS, Biological Industries), in a humidified environment containing 5% CO2 at 37 °C. The cells were seeded onto plates and transduced with lentiviral mCherry‐eGFP‐LC3B (VSVG‐LENTAI‐EF1a‐PuroR‐CMV‐mCherry‐eGFP‐LC3B, Taitool Bioscience) for a duration of 48 h. Following this, 0.04 U of ChABC was incubated with the cells for 3 h, and the cells were subsequently imaged using confocal microscopy.

### Thioflavin S Staining

Thioflavin S (ThS) staining was used to label the senile plaques. Briefly, brain sections were washed three times with PBS and stained with 0.002% ThS (Cat. No. T1892, Sigma‐Aldrich) in the dark for 8 min in 50% ethanol and then washed twice with 50% ethanol and triple with PBS. Afterwards, sections were mounted for imaging or processed for further immunofluorescence.

### Decellularized PFC Tissues

The PFC tissues were collected after denatured ChABC or ChABC treatment for 7 days from WT and 5xFAD mice. The decellularized procedure is similar to previous work.^[^ ^67^ ^]^ Briefly, ≈1 mg of PFC was homogenized (Precellys Evolution Touch homogenizer, Bertin) for 10 s at 6800 rpm in 200 µL of high salt buffer (50 mm Tris‐HCl, 0.25%CHAPS, 25 mm EDTA, 3 m NaCl, pH 7.4) supplemented with protease and phosphatase inhibitors. Homogenate was vortexed at 4 °C for 20 min. Homogenized tissue was spun at 18 000 × g at 4°C for 30 min. The resulting supernatant was removed and saved, and the pellet was further extracted with 500 µL high salt buffer two times with homogenization after each buffer addition. The pellets were dissolved in 80 µL 8 m urea buffer (100 mm ammonium bicarbonate, pH 8.0) supplemented with protease and phosphatase inhibitors at room temperature overnight in the shake cultivation and quantified using BCA Protein Assay Kit (Cat. No. PC0020, Solarbio).

### Western Blotting

After the behavior test, the mice were anesthetized with sodium pentobarbital (60 mg Kg^−1^) and perfused with saline. PFCs were extracted and prepared with RIPA lysis buffer and quantified using BCA Protein Assay Kit (Cat. No. PC0020, Solarbio). Obtained protein lysates were separated by sodium dodecyl sulphate‐polyacrylamide gel electrophoresis (SDS‐PAGE) using 10% gels and blotted onto PVDF membranes. The PVDF membranes were blocked with 10% skim milk in TBST at room temperature on a shaker for 1 hour. The membranes were probed overnight at 4 °C with rabbit monoclonal anti‐GAPDH (D16H11) (1:5000, Cat. No. 5174S, CST), rabbit monoclonal anti‐β Actin (1:30 000, Cat. No. AC038, Abclonal), rabbit monoclonal anti‐fibronectin (1:1500, Cat. No. A12977, Abclonal), rabbit monoclonal anti‐aggrecan (1:1000, Cat. No. A11691, Abclonal), rabbit polyclonal anti‐versican (1:1000, Cat. No. GTX03733, GeneTex), rabbit polyclonal anti‐APP (1:1000, Cat. No. GTX112677, GeneTex), mouse monoclonal anti‐6e10 (1:2000, Cat. No. 80 300, Biolegend), rabbit polyclonal anti‐APP(C‐term) (1:5000, Cat. No. GTX101336, GeneTex), rabbit polyclonal anti‐BACE1(1:1000, Cat. No. K009457P, Solarbio), rabbit monoclonal anti‐EEA1(1:1000, Cat. No. ab2900, Abcam), rabbit monoclonal anti‐Rab7(1:1000, Cat. No. 9367s, Cell Signaling Technology), rabbit polyclonal anti‐Lamp1(1:1000, Cat. No. ab24170, Abcam), rabbit monoclonal anti‐LC3B (1:1000, Cat. No. 43566S, Cell Signaling Technology), rabbit monoclonal anti‐p62 (1:1000, Cat. No. ab109012, Abcam), rabbit monoclonal anti‐ATG16L1(Ser278) (1:500, Cat. No. ab195242, Abcam), rabbit monoclonal ATG16L1 (1:500, Cat. No. 8089S, Cell Signaling Technology), rabbit monoclonal anti‐MERTK (1:2000, Cat. No. ab184086, Abcam) and followed by incubation with HRP‐labeled secondary antibodies and visualized using ECL Western blotting reagents (Cat. No. P90719, Millipore). The bands were detected using the Tanon5200 multi (Tanon), and signals were quantified using ImageJ software (US National Institutes of Health).

### Aβ_1‐42_ Aggregation Preparation and Disaggregation by ChABC

The Aβ_1‐42_ peptide (Cat. No. JT‐92556, Nanjing JT Peptide Biotechnology Co., Ltd.) was solubilized and converted into monomeric form using hexafluoroisopropanol (HFIP) at a concentration of 1 mm. Subsequently, vacuum freeze‐drying was performed, followed by the addition of dimethyl sulfoxide (DMSO) to achieve a final concentration of 1 mm. A concentration of 1 µM Aβ_1‐42_ was introduced into artificial cerebrospinal fluid (ACSF) and subjected to aggregation for 24 h at a temperature of 37 °C with agitation, resulting in the formation of Aβ polymers. Subsequently, the Aβ oligomers were treated with 0.04 U and 0.4 U of ChABC for 24 hours, followed by labeling with ThS for 8 min. Images were captured using a Zeiss800 confocal microscope (Zeiss), and the fluorescence intensity of Aβ_1‐42_ was analyzed utilizing Image J.

### Primary Astrocytes and Microglia Culture

Primary astrocyte and microglia cultures were generated from postnatal 0 to 3 days wild‐type mice. Briefly, mice were decapitated, and the brain was dissected to obtain cortex tissues. Tissues were digested with 0.25% trypsin at 37 °C for 15 min, resuspended as a single cell suspension in DMEM/F‐12 culture medium (Gibco Life Technologies, USA), supplemented with 10% FBS (Gibco Life Technologies, USA) and 1% penicillin‐streptomycin. The cells were filtered using a 70 um cell strainer (Cat. No. 352 350, Falcon) and seeded into culture flasks precoated with poly‐D‐lysine. Cultures were maintained at 37 °C in a humidified 5% CO2 incubator. The culture medium was replaced with a fresh medium every three days. For primary microglia culture, the medium changed into DMEM culture medium (Gibco Life Technologies, USA), supplemented with 20% FBS (Gibco Life Technologies, USA), 1 ng mL^−1^ recombinant mouse FGFb (Cat. No.C044, Novoprotein, China), 1 ng mL^−1^ recombinant mouse beta NGF (Cat. No. C793, Novoprotein, China), and 1% penicillin‐streptomycin. Microglia were removed by gently shaking the culture flasks at 200 rpm for 2 h from the day in vitro (DIV) 10 and plated for downstream experiments. Astrocytes were digested using 0.25% trypsin at DIV 10 and plated for downstream experiments.

### Phagocytosis Assays

The phagocytosis of aggregated Aβ_1‐42_ was analyzed similarly to a previous method.^[^ ^68^ ^]^ Briefly, FITC‐Aβ_1‐42_ (Cat. No.AS‐60479, AnaSpec, Fremont, CA, USA) was aggregated for 48 h at 37 °C with agitation. Primary astrocytes and microglia were plated at 1 × 10^4^ cells per well of 24‐well plates, which contained a 14mm‐diameter coverslip that was precoated with poly‐D‐lysine and cultured overnight. A concentration of 1 µg mL^−1^ FITC‐Aβ_1‐42_ and 0.04 U ChABC were added into the cell medium and incubated for 3 h. For lysosome inhibition assay, 100 nm BafA1 (Cat. No. HY‐100558, MCE) was incubated for 12 h before FITC‐Aβ_1‐42_ and 0.04 U ChABC administration. After FITC‐Aβ_1‐42_ uptake, primary astrocytes and microglia were washed with PBS and fixed with 4% paraformaldehyde for 30 min. Anti‐GFAP antibody was used to label astrocytes, anti‐Iba1 antibody was used to label the microglia, and 4′,6‐diamidino‐2‐phenylindole (DAPI) was used to stain the nuclei. Images were captured using a Zeiss800 confocal microscope (Zeiss), and the fluorescence intensity of FITC‐Aβ_1‐42_ was analyzed utilizing Image J software. The values are represented as the average intensity of intracellular FITC‐Aβ_1‐42_ signal per cell and normalized to the PBS group.

### Isolation of Astrocytes from Adult Mouse PFC

Adult mice were anesthetized with sodium pentobarbital (60 mg Kg^−1^) and perfused transcardially with ice‐cold saline. The PFC tissues were collected after denatured ChABC or ChABC treatment for 7 days from WT and 5xFAD mice and washed with Dulbecco's phosphate‐buffered saline (DPBS, pH 7.4). The adult brain dissociation kit (Cat. No.130‐107‐677, Miltenyi Biotec) was used to obtain single‐cell suspensions from PFC, referring to the manufacturer's instruction. Astrocytes were isolated using anti‐ACSA‐2 MicroBeads (Cat. No.130‐097‐678, Miltenyi Biotec) by using a MACS multistand separator according to the manufacturer's instructions. The isolated astrocytes were pooled from five to six mice for each sample.

### Total RNA Extraction and Quantitative Real‐Time PCR (qRT‐PCR)

After the behavior test, PFC tissues from half brain were taken out for qRT‐PCR and RNA sequencing. Total RNA was extracted using Trizol regent (Cat. No.15596026, Invitrogen) following the manufacturer protocol. The cDNA was synthesized from 2 µg total RNA using a FastKing RT kit (with gDNase) (Cat. No. KR116, TIANGEN). Reactions were performed according to manufacturer protocol. Primer sequences are listed in Table S1. qRT‐PCR was run using SYBR Premix Ex Taq (Tli RNaseH Plus) (Cat. No. RR820A, TaKaRa) with a cycling program of 3 min at 95 °C followed by 40 cycles of 95 °C for 10 s and 60 °C for 30 s on a StepOnePlus Real‐Time PCR Systems (Appliedbiosystems). The differentials of samples were standardized with β‐actin. The mRNA fold changes were normalized by the WT‐denatured ChABC group. Data were analyzed using the delta‐delta Ct method.

### Tandem Mass Tags Proteomic and Bioinformatics Analysis

The PFC tissues from WT‐denatured ChABC, 5×FAD denatured ChABC, and 5×FAD ChABC mice (*n* = 5) were collected for protein preparation as previously described.^[^ ^69^ ^]^ Briefly, the tissues were lysed with PASP lysis buffer (100 mm NH_4_HCO_3_, 8 M Urea, pH 8.0) and quantitated with Bradford assay. Subsequently, the proteins were digested using trypsin and then labeled with TMT reagent (Thermo Scientific). The labeled proteins were delivered by fraction separation and mass spectrometry. The resulting spectra from each run were searched separately against a database using the Proteome Discoverer 2.4 (PD 2.4, Thermo). Peptide Spectrum Matches (PSMs) with a credibility of more than 99% were identified PSMs. The identified protein contains at least 1 unique peptide. The identified PSMs and protein were retained and performed with FDR no more than 1.0%. All of these experimental procedures were conducted by Novogene Co. Ltd (Tianjin, China). The proteins exhibiting a significant difference in quantitation between the experimental and control groups, with a *P* value < 0.05 and a fold change ≥ 1.2 or ≤ 0.83, were established as the threshold for defining differentially expressed proteins (DEP) using the limma R package.

All of the figures were obtained using R packages. Gene Ontology representation of gene enrichment was generated by the “clusterProfiler” (4.8.1) and “org.Mm.eg.db” (3.17.0) package of R. Heatmap representation of gene expression was generated by the “pheatmap” package of R (https://CRAN.R‐project.org/package=pheatmap).

### RNA‐Sequencing and Bioinformatics Analysis

The PFC tissues or astrocytes from 5×FAD mice and WT mice were collected for RNA preparation (the same samples as qPCR). mRNA library construction and sequencing were performed by Novogene Co. Ltd (Tianjin, China) and Wekemo Technology Co., Ltd. (Shenzhen, China). The mapped reads of each sample were assembled using StringTie (v1.3.3b). The feature Counts v1.5.0‐p3 was used to count the reads mapped to each gene. Then, the FPKM of each gene was calculated based on the length of the gene and the read count mapped to this gene.

Differential gene expression analysis of two groups (five biological replicates per group) was performed using the DESeq2 package (1.26.0). The resulting P‐values were adjusted using Benjamini and Hochberg's approach for controlling the false discovery rate. *P* Value < 0.05 and |log2(foldchange)| > = 1 were set as the threshold for significant differential by R packages. All of the figures were obtained with R packages. Gene Ontology representation of gene enrichment was generated by the “clusterProfiler” (4.8.1) and “org.Mm.eg.db” (3.17.0) package of R. Heatmap representation of gene expression was generated by the “pheatmap” package of R (https://CRAN.R‐project.org/package=pheatmap).

### Transmission Electron Microscope

The mPFC were taken out after 7 days under denatured ChABC or ChABC injection into mPFC, cut into 3 × 1 × 1 mm small pieces, and fixed with 2.5% glutaraldehyde in 0.01 mol L^−1^ sodium phosphate buffer (pH 7.4), followed by 1% osmium tetroxide. After dehydration, thin sections were cut using a Leica EM UC7 Ultramicrotome (Leica) and stained with uranyl acetate and lead citrate. The ultrastructural analysis was performed using a HITACHI HT 7800 120kv electron microscope (Hitachi High‐Tech).

### Statistics

All data analysis and statistics were calculated with GraphPad Prism 9.0 (GraphPad Software) and Origin 8.5 software. Two groups were compared using an unpaired two‐sided Student's *t*‐test. In more than two experimental groups, data were analyzed by a one‐way ANOVA test. When the one‐way ANOVA tests were statistically significant, Bonferroni's and Dunn's multiple‐comparison post hoc analyses, respectively, were used to compare the differences between individual groups. *P* values less than 0.05 were considered statistically significant and are indicated by *P < 0.05, **P < 0.01, and ***P < 0.001 in the figures. Data are shown as mean ± standard errors (SEM). All statistical data are shown in Table S2 (Supporting Information).

## Conflict of Interest

The authors declare no conflict of interest.

## Author Contributions

Q.H.Y., C.X.Y., and Y.H.S. contributed equally to this work. Z.T.B. and Z.Q.Y. conceived and directed the project. Q.H.Y. and Z.T.B. designed the experiments. Q.H.Y., C.X.Y., and Y.H.S. performed the experiments, analyzed the data, and wrote the manuscript. Z.X., L.Y., and M.J. provided assistance with animal behavioral tests. B.N.C. and Z.Y.Y. provided assistance with the bioinformatic analysis of the RNA sequence and proteomics. J.J.N., Y.Y.W., X.L., and S.X. contributed to data analysis. All authors discussed and commented on the manuscript.

## Supporting information

Supporting Information

Supplemental Data 1

Supplemental Data 2

Supplemental Data 3

Supplemental Data 4

## Data Availability

Mouse proteomics data are available in the PRIDE database (http://www.proteomexchange.org) with accession numbers of PXD046784. The RNA sequence data utilized in this manuscript can be accessed in the NCBI Sequence Read Archive, specifically under the accession number PRJNA1036358. All data needed to evaluate the conclusions in the paper are present in the paper and/or the Supplementary Materials. Additional data related to this paper may be requested from the authors.
