# Supplementary material for: Extracellular Matrix Remodeling Alleviates Memory Deficits in Alzheimer's Disease by Enhancing the Astrocytic Autophagy‐Lysosome Pathway
Source: Adv Sci (Weinh). 2024 Jun 17;11(31):2400480. doi: 10.1002/advs.202400480 (PMC11336928; doi:10.1002/advs.202400480)
Supplement: Supplementary file 1 — Supporting Information [file ADVS-11-2400480-s001.docx]

Supplementary Materials for

**Extracellular matrix remodeling alleviates memory deficits in Alzheimer’s disease by enhancing the astrocytic autophagy-lysosome pathway**

Qinghu Yang *et al*

* Corresponding author. Email: zqyuan@bmi.ac.cn; ztbai@yau.edu.cn.

**This PDF file includes:**

Figs. S1 to S12

Tables S1 and S2

Data S1 to S4


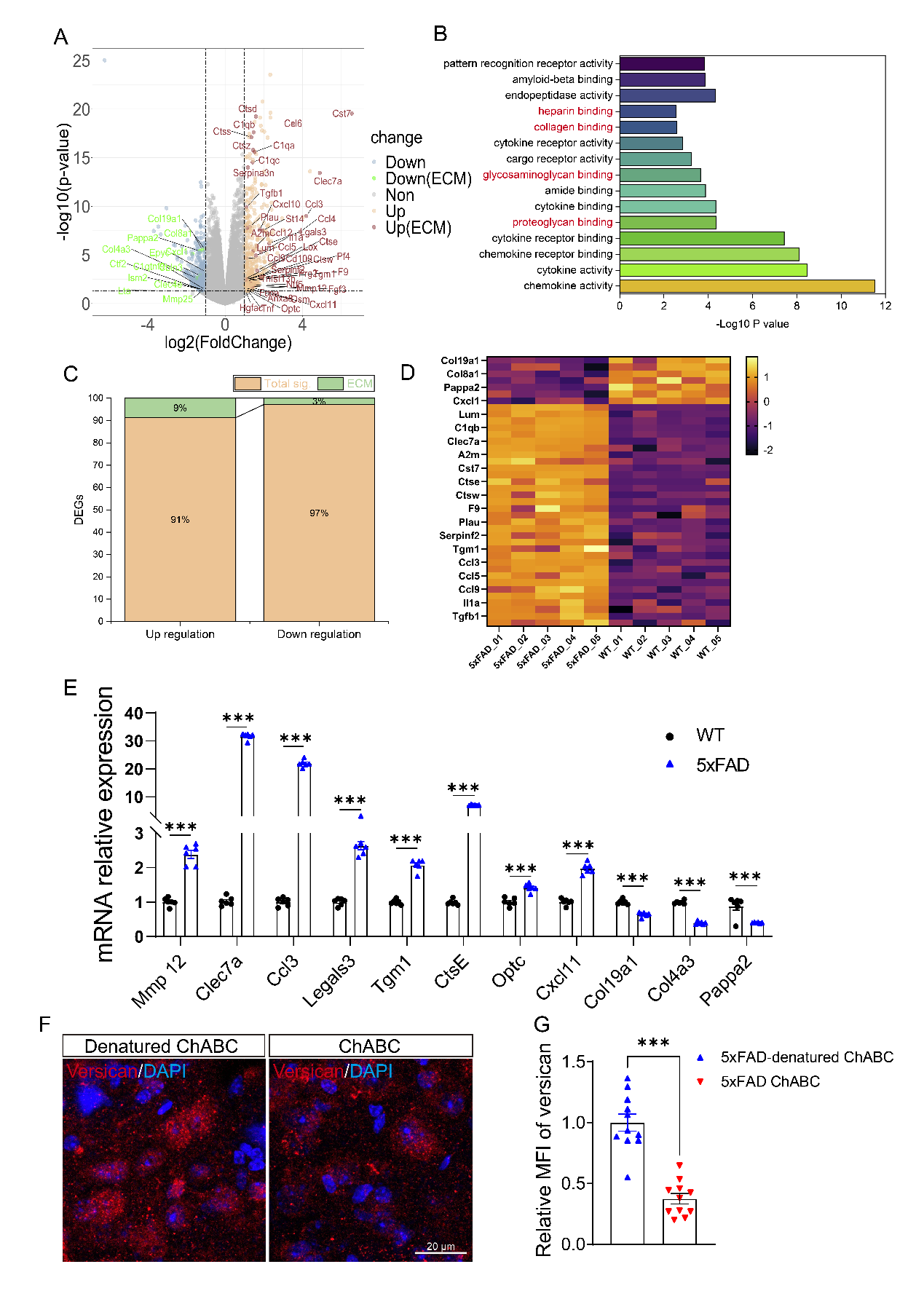


**Figs. S1. Differential dynamic expression of ECM genes in PFC of WT and AD mice**

**(A)** The volcano plot showed the upregulation and downregulation genes in the 5xFAD mice versus WT mice (n = 5). The green dots represent ECM downregulation genes, and the brown dots represent ECM upregulation genes. **(B)** GO enrichment of differential expression genes. **(C)** The ratio of ECM genes in the differential expression genes. **(D)** Heatmap represents the up-and-down-expression genes of ECMs in the 5xFAD mice versus WT mice. **(E)** The mRNA expression level of ECM association DEGs. **(F)** Represent image of versican in mPFC of 5xFAD mice after ECM remodeling. The scale bar is 20 μm. **(G)** Quantification of the fluorescence intensity of versican in mPFC of 5xFAD mice after ECM remodeling. Data in (E) and (G) are means ± SEM (numbers in bars show biological replicates/ROIs). Statistical analyses were performed by unpaired two-sided Student’s t-test, ****P*<0.001.


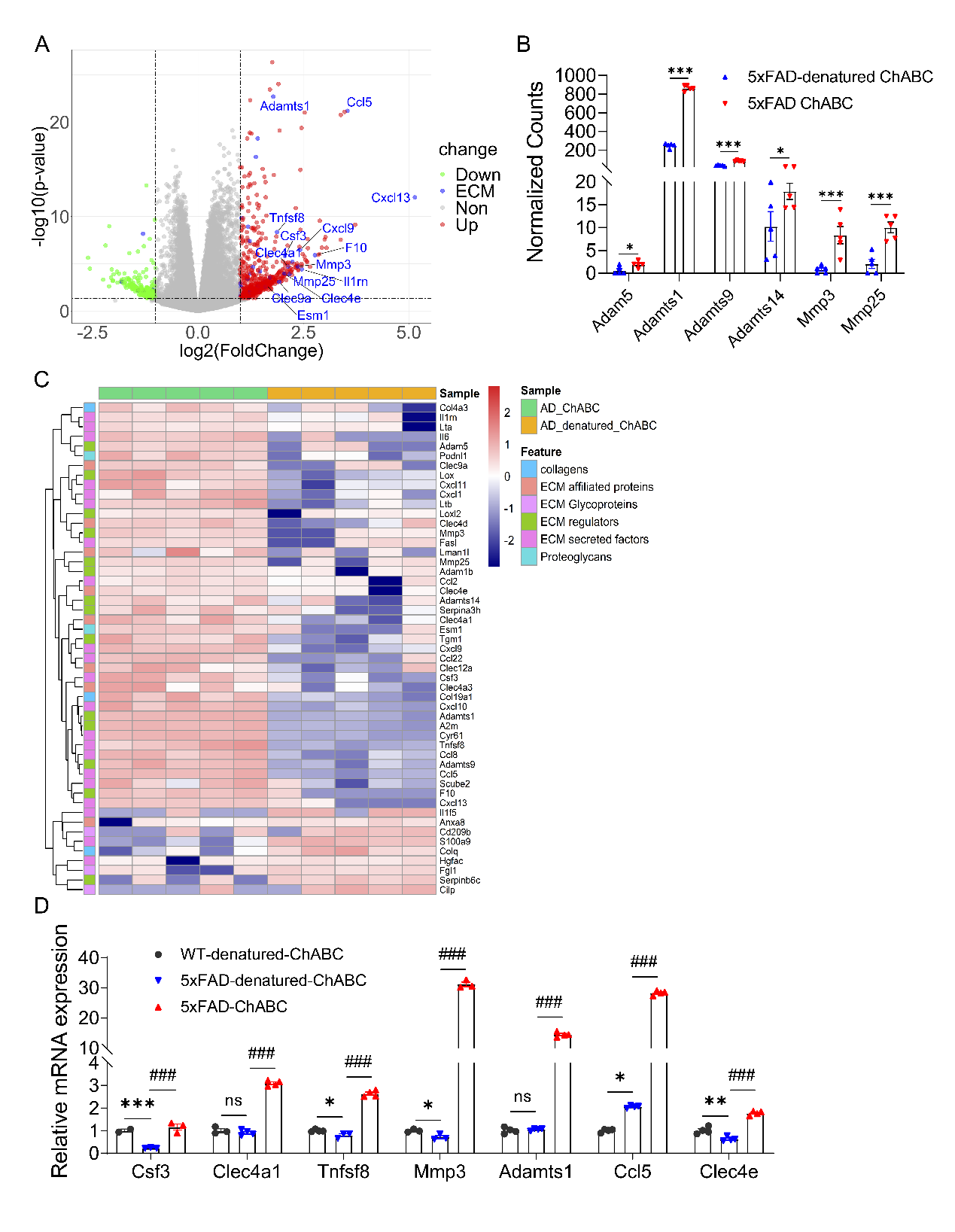


**Figs. S2. ChABC promotes ECM remodeling in mPFC of 5xFAD mice**

**(A)** The volcano plot showed the upregulation and downregulation genes in the 5xFAD mice after ChABC or denatured ChABC administration (n = 5). The blue dots represent ECM genes. **(B)** The normalized counts of ECM remodeling enzymes. **(C)** Heatmap represents the up-and-down-expression genes of ECMs in different functional features in the 5xFAD mice after ChABC or denatured ChABC administration. **(D)** The mRNA expression level of ECM association DEGs. Data in (B) and (D) are means ± SEM (numbers in bars show biological replicates). Statistical analyses were performed by Two-way ANOVA with Bonferroni’s multiple comparisons test, **P*<0.05, ***P*<0.01, ****P*<0.001, ###*P*<0.001, ns, no significant.


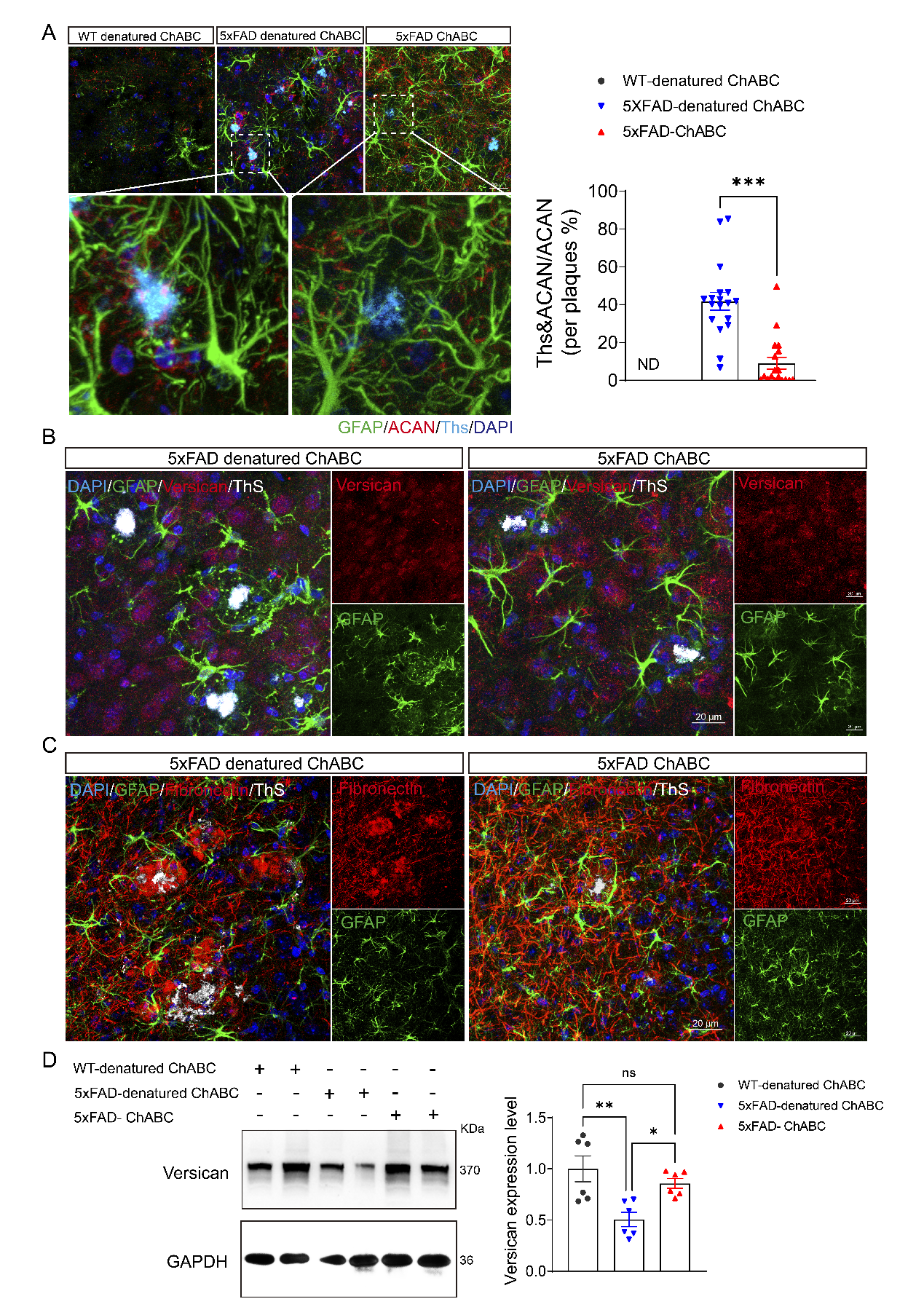


**Figs. S3. ChABC promotes ECM remodeling to reduce the entanglement of ECMs around Aβ plaques**

**(A)** Immunofluorescent images of GFAP (green), ACAN (red), and ThS (cyan) in mPFC from 5xFAD mice and WT mice after denatured ChABC or ChABC administration. The scale bar is 20 μm. Quantification of the percentage of ACAN per plaque to total ACAN in the WT-denatured ChABC, 5xFAD-denatured ChABC and 5xFAD-ChABC mice. **(B)** Immunofluorescent images of Versican (red), GFAP (green), and Ths (white) in mPFC from 5xFAD mice after denatured ChABC or ChABC administration. The scale bar is 20 μm. **(C)** Immunofluorescent images of Fibronectin (red), GFAP (green), and Ths (white) in mPFC from 5xFAD mice after denatured ChABC or ChABC administration. The scale bar is 20 μm. **(D)** Western blotting analysis of versican and GAPDH in PFC from WT denatured-ChABC, 5xFAD denatured-ChABC, and 5xFAD-ChABC mice. The right panel is the qualification of versican expression level. Data in (A) and (D) are means ± SEM (numbers in bars show cells/biological replicates). Statistical analyses were performed by unpaired two-sided Student’s t-test (A),or One-way ANOVA with Bonferroni’s multiple comparisons test, **P*<0.05, ***P*<0.01, ****P*<0.001, ns, no significant.


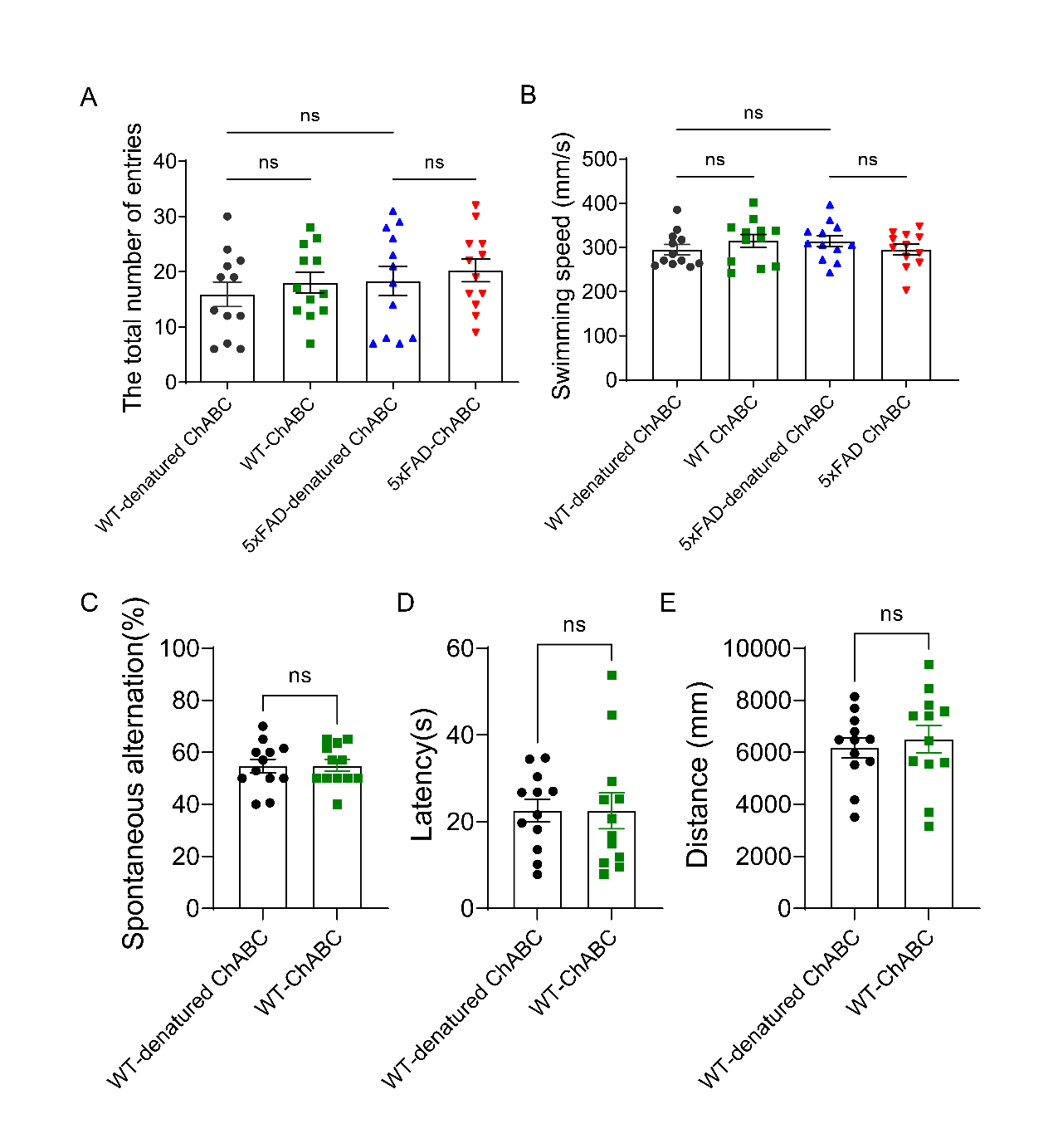


**Figs. S4. ECM remodeling does not alter the short-term memory performance of WT mice.**

**(A)** The total number of entries of Y maze from WT and 5xFAD mice after ECM remodeling. **(B)** The swimming speed of the DMP task from WT and 5xFAD mice after ECM remodeling. (**C**) The percentage of spontaneous alternation among WT-denatured ChABC mice and WT-ChABC mice. **(D)** The escape latency between the two groups. **(E)** The swimming distance between the two groups. Data in (A) to (E) are means ± SEM (numbers in bars show biological replicates). Statistical analyses were performed with One-way ANOVA with Bonferroni’s multiple comparisons test (A and B), or unpaired two-sided Student’s t-test, ns, no significant.


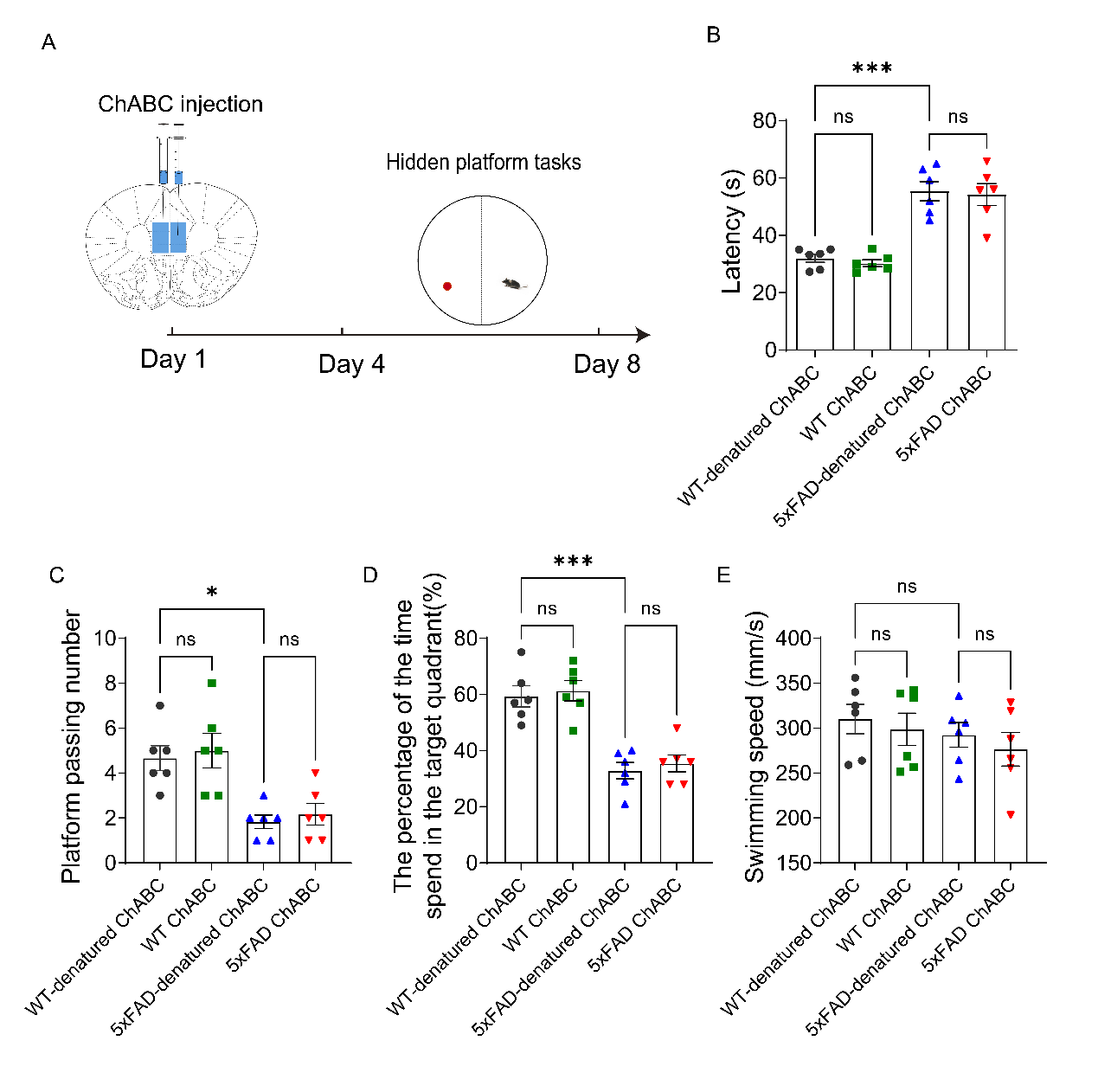


**Figs. S5. ECM remodeling does not alter the long-term memory performance of AD mice.**

**(A)** The schematic illustration of the ChABC injection and hidden platform task. The ChABC was injected on day 1, and the hidden platform task was performed from day 4 to day 8. **(B)** The escape latency of hidden platform task from WT and 5xFAD mice after ECM remodeling. **(C)** The platform passing number of hidden platform tasks from WT and 5xFAD mice after ECM remodeling. **(D)** The percentage of time spent in the target quadrant of hidden platform task from WT and 5xFAD mice after ECM remodeling. **(E)** The swimming speed of hidden platform task from WT and 5xFAD mice after ECM remodeling. Data in (B) to (E) are means ± SEM (numbers in bars show biological replicates). Statistical analyses were performed with One-way ANOVA with Bonferroni’s multiple comparisons test, **P*<0.05, ****P*<0.001, ns, no significant.


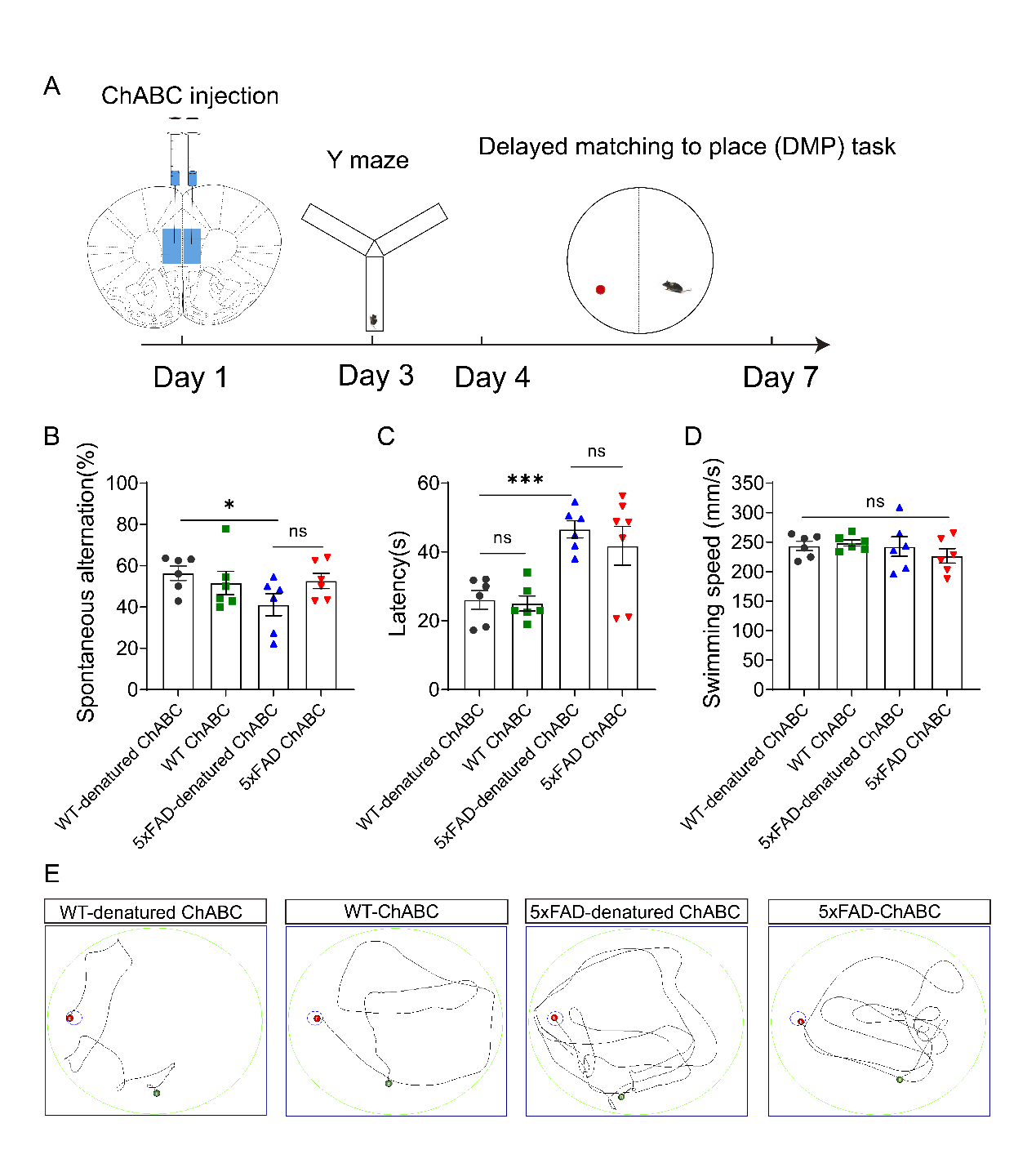


**Figs. S6. ECM remodeling does not alleviate the short-term spatial memory defects in 9-month 5xFAD mice.**

**(A)** The schematic illustration of the ChABC injection, the Y maze, and delayed matching to place task. The ChABC was injected on day 1, the Y maze was performed on day 3, and DMP was performed from day 4 to day 7. **(B)** The percentage of spontaneous alternation from WT and 5xFAD mice after ECM remodeling. **(C)** The escape latency from WT and 5xFAD mice after ECM remodeling. **(D)** The swimming speed from WT and 5xFAD mice after ECM remodeling. **(E)** The swimming pathway for the WT-denatured ChABC mice, WT ChABC mice, 5xFAD-denatured ChABC mice, and 5xFAD-ChABC mice. Data in (B) to (D) are means ± SEM (numbers in bars show biological replicates). Statistical analyses were performed with One-way ANOVA with Bonferroni’s multiple comparisons test, **P*<0.05, ****P*<0.001, ns, no significant.


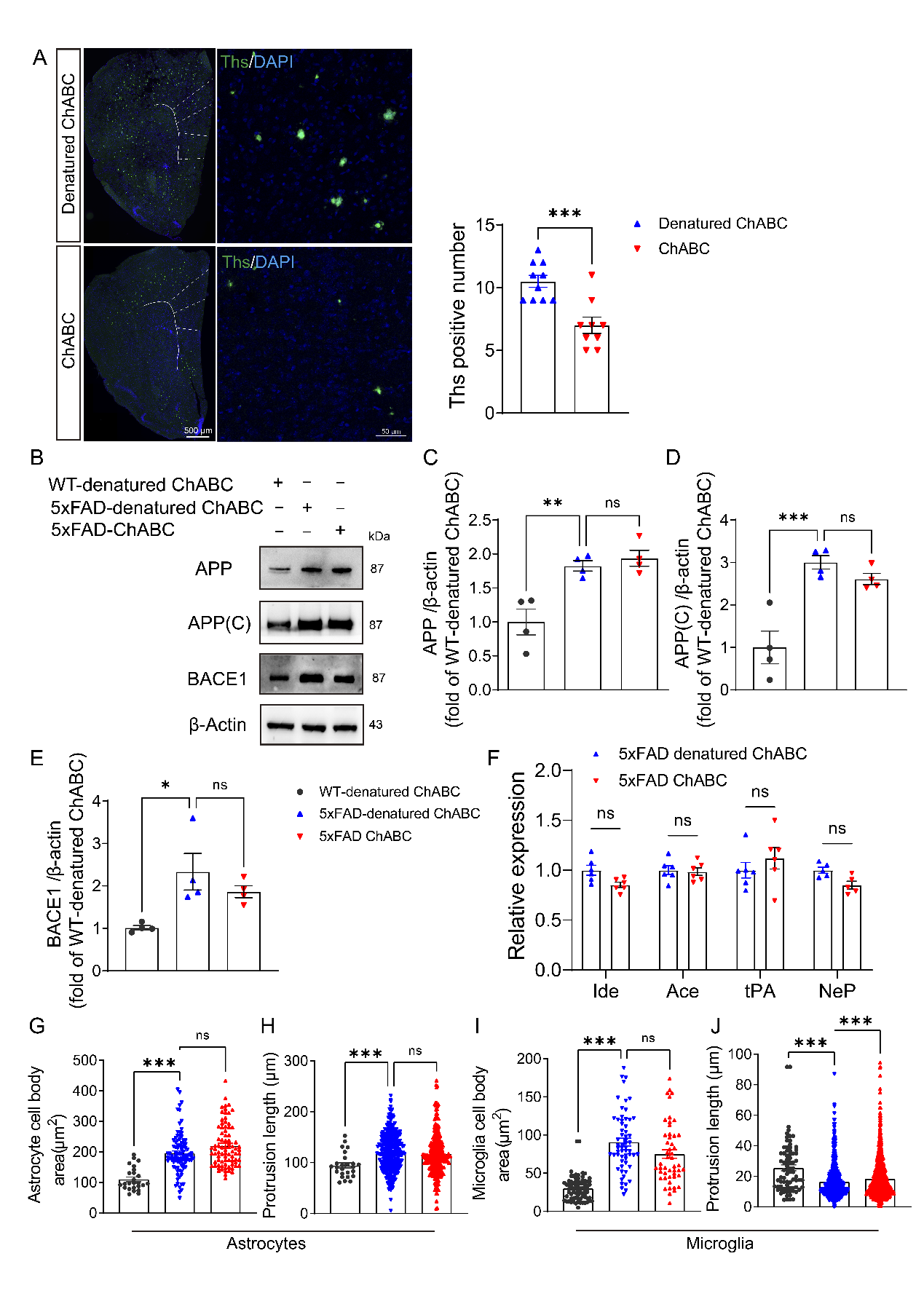


**Figs. S7. ECM remodeling degrades Aβ plaques but does not alter the procession of Aβ production.**

**(A)** The left panel represent the image of Ths in mPFC in the denatured ChABC and ChABC groups in AD mice. The scale bar is 500 μm or 50 μm. Right panel, quantification of the number of Ths positive puncta in the denatured ChABC and ChABC groups (n=6, unpaired two-sided Student’s t-test, ***P<0.001). **(B)** Western blotting analysis of the expression of APP, APP(C), BACE1, and β-actin in PFC in WT-denatured ChABC mice, 5xFAD-denatured ChABC mice, and 5xFAD ChABC mice groups. **(C)** Quantification of the expression level of APP in the western blotting shown in (B). **(D)** Quantification of the expression level of APP(C) in the western blotting shown in (B). **(E)** Quantification of the expression level of BACE1 in the western blotting shown in (B). **(F)** Relative expression of Aβ degradation enzymes (*Ide, Ace, tPA*, and *NeP*) in mPFC from 5xFAD mice after ECM remodeling. **(G)** and **(H)** The astrocyte cell body area (G) and protrusion length (H) in the mPFC from WT-denatured ChABC, 5xFAD-denatured ChABC, and 5xFAD ChABC mice. **(I)** and **(J)** The microglia cell body area (I) and protrusion length (J) in the mPFC from WT-denatured ChABC, 5xFAD-denatured ChABC, and 5xFAD ChABC mice. Data in (A), (C) to (J) are means ± SEM (numbers in bars show biological replicates/cells). Statistical analyses were performed by unpaired two-sided Student’s t-test (A), One-way ANOVA with Bonferroni’s multiple comparisons test in (C) to (E) and (G) to (J), or Two-way ANOVA with Bonferroni’s multiple comparisons test (F), **P*<0.05, ****P*<0.001, ns, no significant.


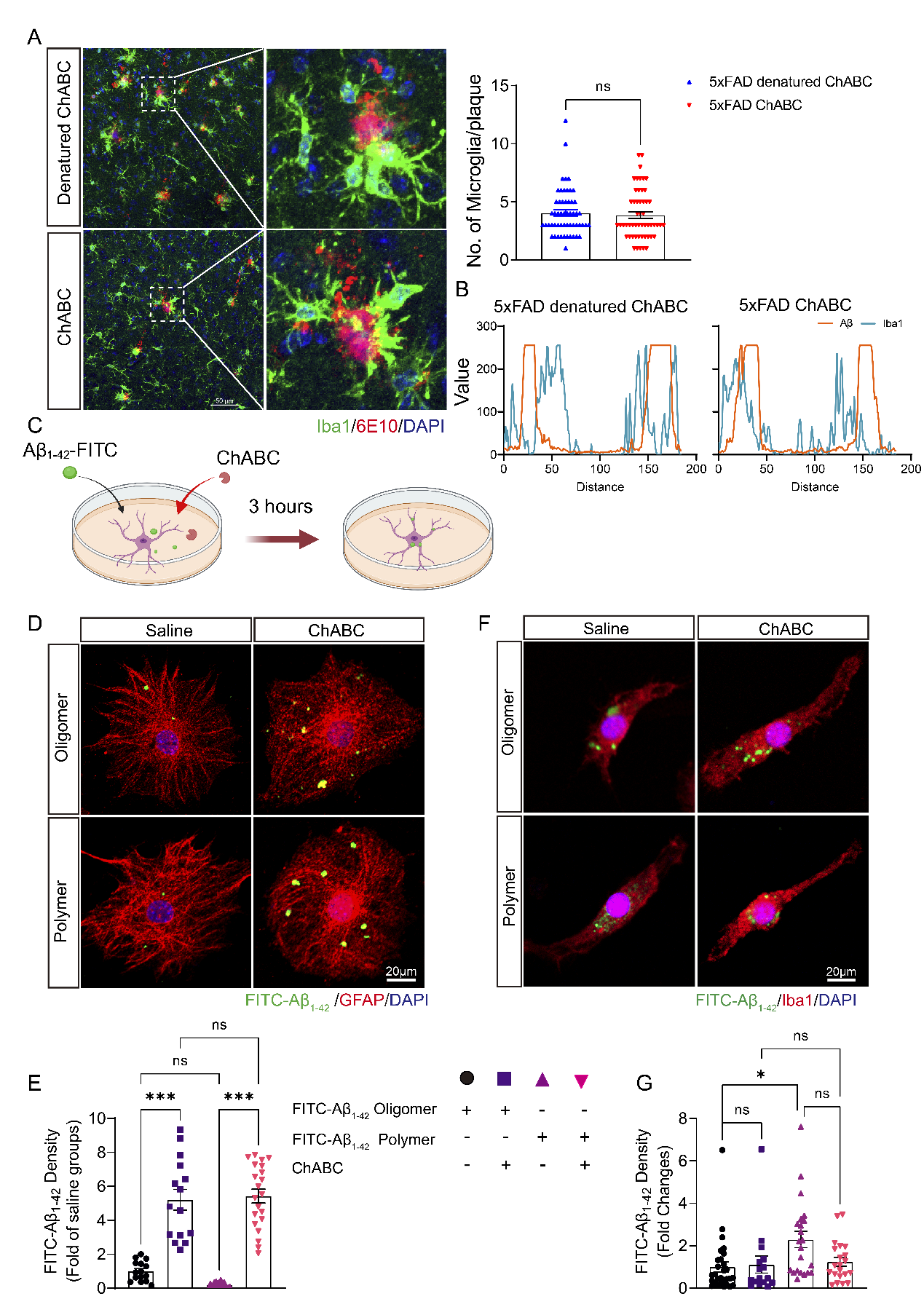


**Figs. S8. ECM remodeling promote astrocytic but not microglial Aβ phagocytosis**

**(A)** Immunofluorescent images of 6E10 (red) and Iba1 (green) in mPFC from 5xFAD mice after denatured ChABC or ChABC administration. The scale bar is 20 μm. The right panel is the quantification of the number of microglia per plaque in the 5xFAD-denatured ChABC and 5xFAD-ChABC mice. **(B)** Co-localization analysis of 6E10 and Iba1 from 5xFAD-denatured ChABC mice (left) and 5xFAD-ChABC mice (right). **(C)** The FITC-Aβ_1-42_ phagocytic assay in primary astrocytes or microglia after saline or 0.04U ChABC treatment. The oligomer FITC-Aβ_1-42_, polymer FITC-Aβ_1-42_ and ChABC were incubated for 3 hours. **(D)** The image represent the phagocytosis of FITC-Aβ_1-42_ by astrocytes. The scale bar is 20 μm. **(E)** The quantification of FITC-Aβ1-42 density in astrocytes. **(F)** The phagocytosis of FITC-Aβ_1-42_ by microlia. The scale bar is 20 μm. **(G)** The quantification of FITC-Aβ1-42 density in microlia. Data in (A), (E) and (G) are means ± SEM (numbers in bars show cells). Statistical analyses were performed by unpaired two-sided Student’s t-test (A), One-way ANOVA with Bonferroni’s multiple comparisons test in (E) and (G), **P*<0.05, ****P*<0.001, ns, no significant.


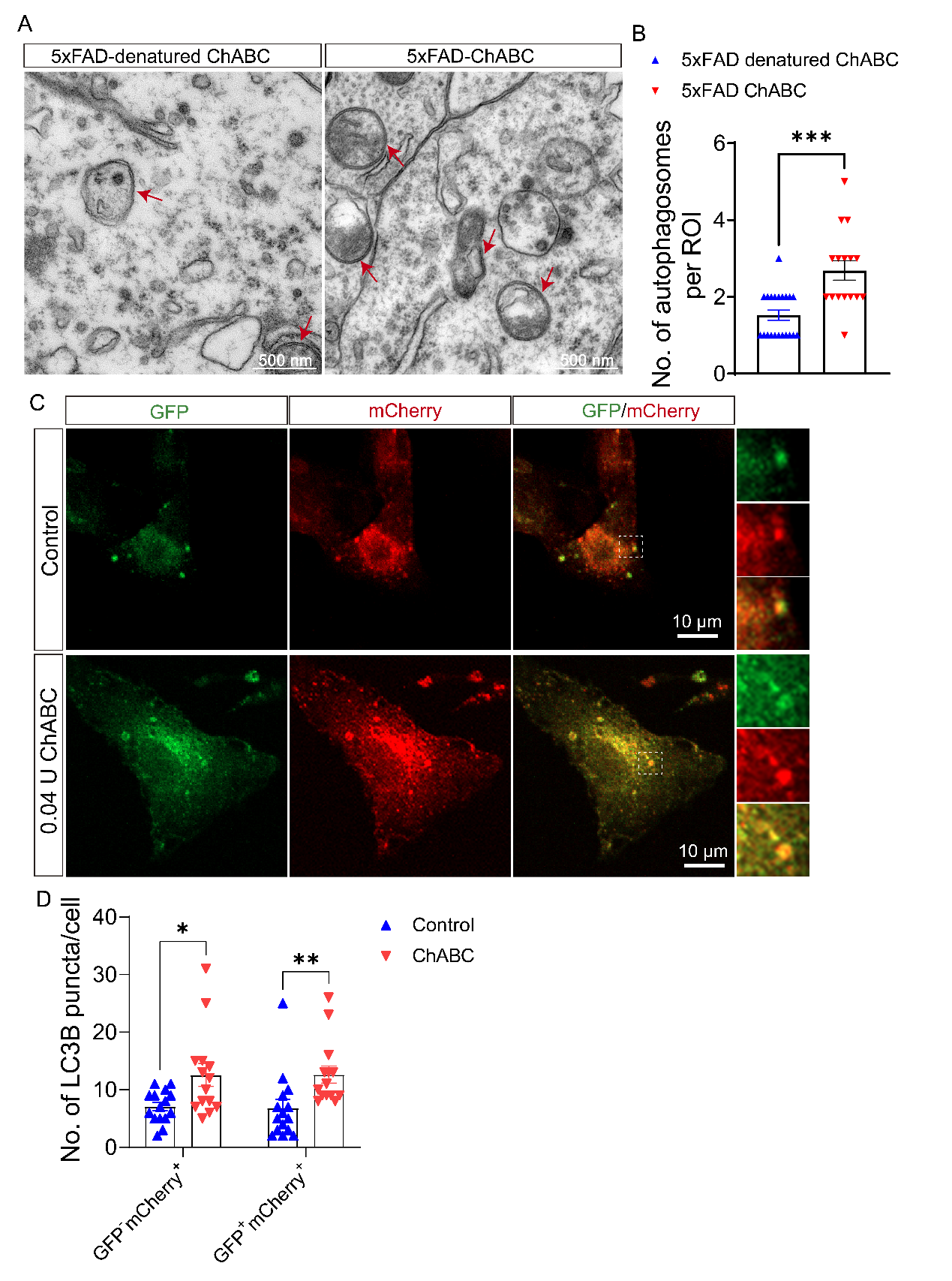


**Figs. S9. ECM remodeling enhanced astrocytical autophagic flux**

**(A)** Transmission electron microscope images of the autophagosome (red arrow) in PFC from 5xFAD mice after ECM remodeling. The scale bar is 500 nm. **(B)** The number of autophagosome per ROI in mPFC from 5xFAD mice after ECM remodeling. **(C)** Confocal image of the eGFP-mCherry-LC3B signal in Gl261 cells under 0.04 U ChABC incubation for 3 hours. The scale bar is 10 μm. (D) Quantification of the GFP^-^mCherry^+^ and GFP^+^mCherry^+^ positive LC3B puncta in (C). Data in (B) and (D) are means ± SEM (numbers in bars show cells). Statistical analyses were performed by unpaired two-sided Student’s t-test in (B), Two-way ANOVA with Bonferroni’s multiple comparisons test in (D), **P*<0.05,***P*<0.01, ****P*<0.001, ns, no significant.


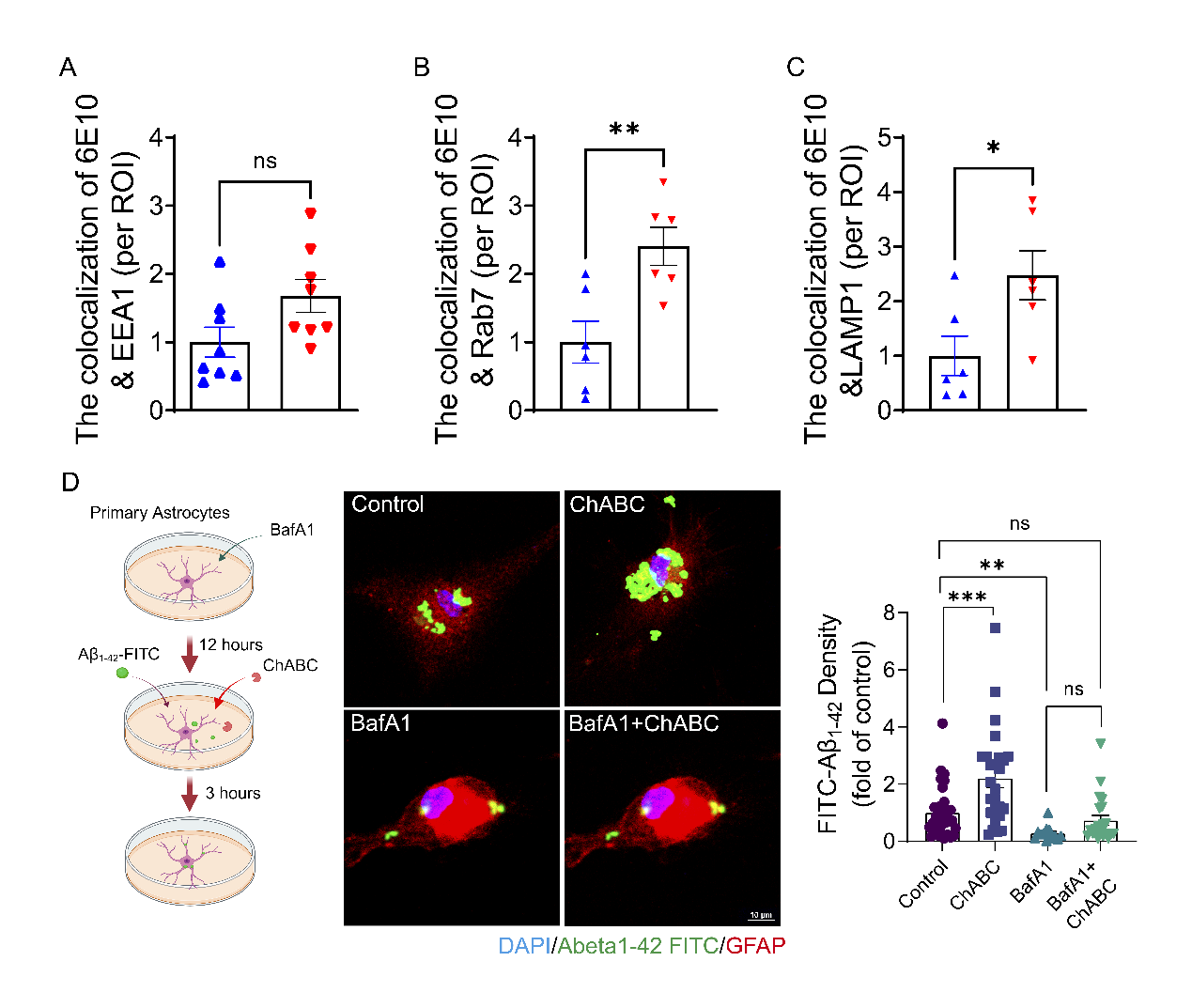


**Figs. S10. ECM remodeling activates astrocyte autophage-lysosome pathway to clear Aβ plaques**

**(A)** to **(C)** Quantification of the colocalization of 6E10 and EEA1 (A), 6E10 and Rab7 (B), and 6E10 and Lamp1 (C) in mPFC from 5xFAD mice after ECM remodeling. **(D)** The FITC-Aβ_1-42_ phagocytic assay in primary astrocytes after saline or BafA1 treatment. The polymer FITC-Aβ_1-42_ and 0.04 U ChABC were incubated for 3 hours (left). The image represent the phagocytosis of FITC-Aβ_1-42_ by astrocytes. The scale bar is 10 μm (Middle). The quantification of FITC-Aβ1-42 density in astrocytes (Right). Data in (A) to (D) are means ± SEM (numbers in bars show biological replicates/cells). Statistical analyses were performed by unpaired two-sided Student’s t-test in (A) to (C), One-way ANOVA with Bonferroni’s multiple comparisons test in (D), **P*<0.05, ***P*<0.01, ****P*<0.001, ns, no significant.


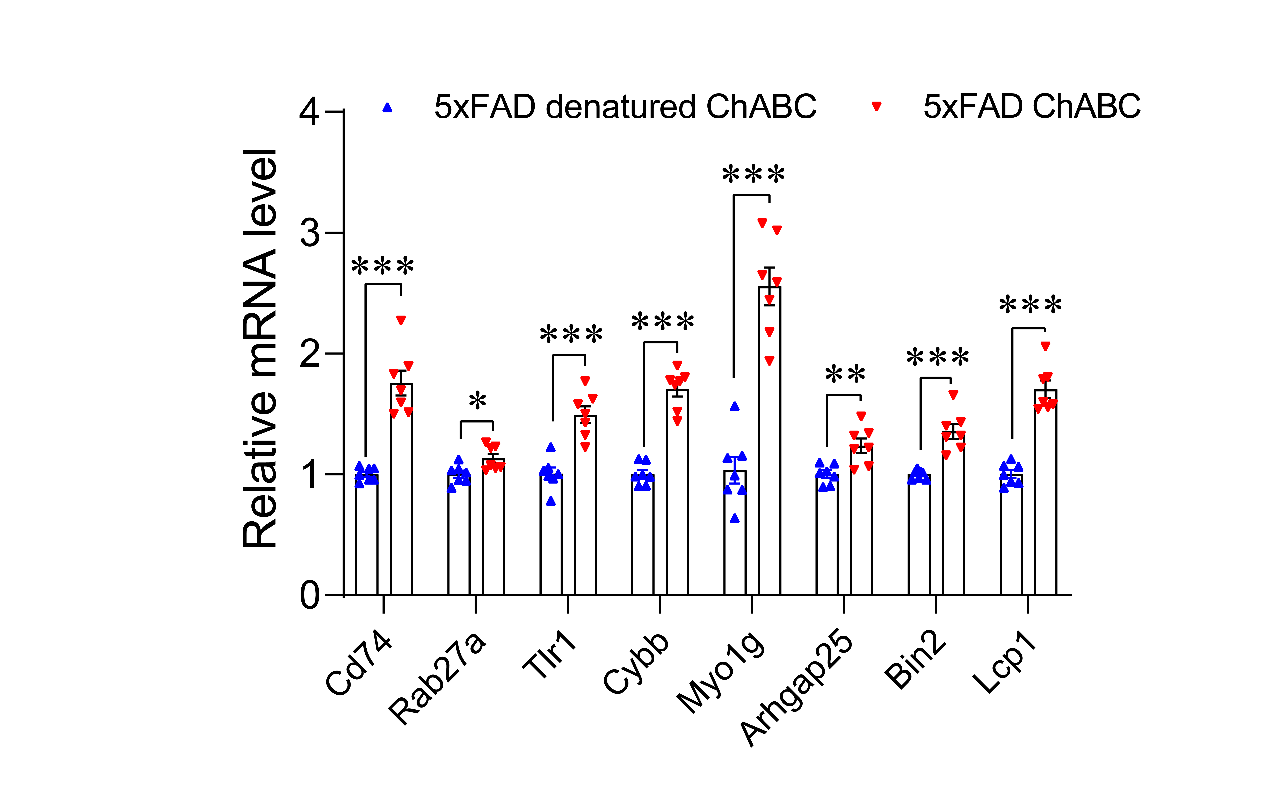


**Figs. S11. Real-time PCR analysis of differential genes in astrocytes from 5xFAD mice under ECM remodeling.**

Graph data is expressed as mean ± SEM (numbers in bars show biological replicates). Statistical analyses were performed with Two-way ANOVA with Bonferroni’s multiple comparisons test, *P<0.05, **P<0.01, ***P<0.001.


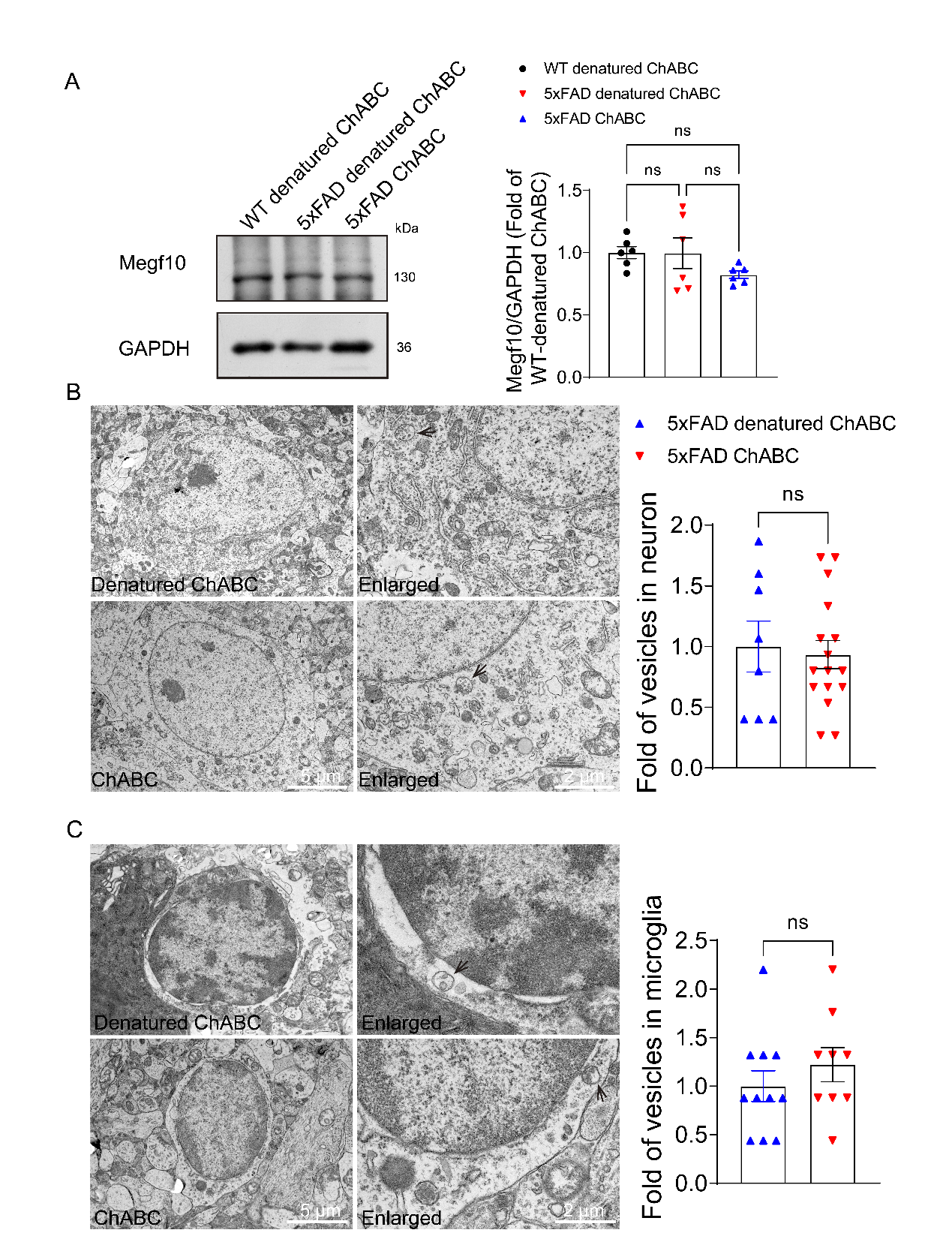


**Figs. S12. Transmission electron microscope images of phagocytosis-related vesicles in neuron and microglia under ECM remodeling in AD mice**

**(A)** Western blotting analysis of megf10 and GAPDH in PFC from WT denatured-ChABC, 5xFAD denatured-ChABC, and 5xFAD-ChABC mice. The right panel is the qualification of megf10 expression level. **(B)** Image represents the vesicles in neurons in PFC from 5xFAD mice after denatured ChABC or ChABC administration. The right panel is the qualification of the number of vesicles per neuron (3 mice per group, 3-6 neurons per mouse). The left scale bar is 5 μm, and the right scale bar is 2 μm. **(C)** The image represents the vesicles in microglia in PFC from 5xFAD mice after denatured ChABC or ChABC administration. The right panel is the qualification of the number of vesicles per microglia (3 mice per group, 3-6 microglia per mouse). The left scale bar is 5 μm, and the right scale bar is 2 μm. Graph data are expressed as mean ± SEM. Statistical analyses were performed by One-way ANOVA with Bonferroni’s multiple comparisons test (A), or unpaired two-sided Student’s t-test in (B) and (C), ns, no significance.

**Table S1. Primer sequences in this work.**

| **Name** | **Forward primer** | **Reverse primer** |
| --- | --- | --- |
| Mmp12 | CACACTTCCCAGGAATCAAGCC | TTTGGTGACACGACGGAACAGG |
| Mmp3 | CTCTGGAACCTGAGACATCACC | AGGAGTCCTGAGAGATTTGCGC |
| Adamts1 | GAAGGCAAACGAGTCCGCTACA | TTGGGTGTCCACTCTACAGTGG |
| Clec7a | CCAGCTAGGTGCTCATCTACTG | CCTTCACTCTGATTGCGGGAAAG |
| Clec4a1 | GTGACACTGCATCCTGGAGTAAG | ACGAGGGTTCAGAGTGTTGGTG |
| Clec4e | TTTTAAAAAGAGGGCCAAGGATTCA | AAGCATCCTCTCTCTGTGTGG |
| Col19a1 | GGAAGAACTGGACATCCTGGTC | ATGCCGTTTCCCTCATTGAGCG |
| Col4a3 | GATGGGCTATCCTGGAACCACT | TTCTCTCCTCGTTCGCCTTTTGG |
| Ccl3 | ACTGCCTGCTGCTGCTCTCCTACA | ATGACACCTGGCTGGGAGCAAA |
| Ccl5 | CCTGCTGCTTTGCCTACCTCTC | ACACACTTGGCGGTTCCTTCGA |
| Cxcl11 | CCGAGTAACGGCTGCGACAAAG | CCTGCATTATGAGGCGAGCTTG |
| Lgals3 | AACACGAAGCAGGACAATAACTGG | GCAGTAGGTGAGCATCGTTGAC |
| Tgm1 | ATCTGCCCTCAGGCTTTGATGG | CGTTCTTGACGGACTCCACAGA |
| CtsE | TCCATCGCAGTCCGACACATAC | GACACTTTCTCCAAACTGCTGGC |
| Optc | CCTGAGCATAGAGGACTACAACG | GTCTTCTGAGCCACAGTGCTAG |
| Pappa2 | TTGGCTGCCTCCAATCAGTGGA | CCTCTGGAGTCCAGTAACCTGA |
| Tnfsf8 | AGAGGATCTCTTCTGTACCCTGA | ATGAGTCCGTGGATGGTGCCAT |
| Csf3 | ATCCCGAAGGCTTCCCTGAGTG | AGGAGACCTTGGTAGAGGCAGA |
| Cd74 | GCTGGATGAAGCAGTGGCTCTT | GATGTGGCTGACTTCTTCCTGG |
| Rab27a | GAGCAAAGTTTCCTCAATGTCCG | CTTTCACTGCCCTCTGGTCTTC |
| Bin2-R | GCCAAGATGGCTAAGGCAGA | AGGCCCTTCACCACAAAGAC |
| Lcp1 | TCTGTGCCAGACACGATTGACG | GAGGCAGAGTTCAGAGCCAAGT |
| Arhgap25 | CAGGACAACCTTGTGAAGCAGC | TGACTCCAAGGAACCACAGGCT |
| Myo1g | TCCAGGACTTCAAACGGCTGCT | GACGCTTGGTCACTTCCGTGAT |
| Tlr1 | GGTAGCAAGAGAAGTGGTGGAG | CGATGGTGACAGTCAGCAGAAC |
| Cybb | TGGCGATCTCAGCAAAAGGTGG | GTACTGTCCCACCTCCATCTTG |
| tPA | GTTACACAGCGTGGAGGACCAA | CACGTCAGCTTTCGGTCCTTCA |
| Ide | CAAACCTCTCCTTCCAAGTCAGC | TGTTCTCCGAGGTGCTCTGCAT |
| Nep | CAGCCGAAACTACAAGGAGTCC | CATAAAGCCTCCCCACAGCATTC |
| Ace | TCAGCGGAATGGCGAAGTCCTA | CGGTCATACTCTTCCACGAACC |

**Table S2. Statistic data for graphs in Figures.**

| **Figure** | **Panel** | **Comparison** | **P-value** | **t (q)** | **DF(df)** |
| --- | --- | --- | --- | --- | --- |
| 1 | F | 5xFAD-denatured ChABC vs. WT-denatured ChABC | 0.09 | 2.404 | 15 |
| 1 | F | 5xFAD-ChABC vs. 5xFAD-denatured ChABC | <0.001 | 4.884 | 15 |
| 1 | F | 5xFAD-denatured ChABC vs. WT-denatured ChABC | 0.08 | 2.48 | 15 |
| 1 | G | WT-denatured ChABC vs. 5xFAD-denatured ChABC | >0.99 | 0.3057 | 15 |
| 1 | G | WT-denatured ChABC vs. 5xFAD-ChABC | 0.002 | 4.381 | 15 |
| 1 | G | 5xFAD-denatured ChABC vs. 5xFAD-ChABC | 0.003 | 4.075 | 15 |
| 2 | B | 5xFAD-denatured ChABC vs. WT-denatured ChABC | 0.004 | 3.262 | 22 |
| 2 | B | 5xFAD ChABC vs. 5xFAD-denatured ChABC | 0.006 | 3.05 | 22 |
| 2 | C | 5xFAD-denatured ChABC vs. WT-denatured ChABC | 0.002 | 3.53 | 22 |
| 2 | C | 5xFAD-ChABC vs. 5xFAD-denatured ChABC | 0.001 | 3.853 | 22 |
| 2 | D | 5xFAD-denatured ChABC vs. WT-denatured ChABC | <0.001 | 5.333 | 22 |
| 2 | D | 5xFAD-ChABC vs. 5xFAD-denatured ChABC | 0.002 | 3.904 | 22 |
| 3 | B | 5xFAD-ChABC vs. 5xFAD-denatured ChABC | 0.012 | 3.051 | 10 |
| 3 | C | 5xFAD-ChABC vs. 5xFAD-denatured ChABC | 0.004 | 3.258 | 20 |
| 3 | D | Over 25 μm :5xFAD-ChABC vs. 5xFAD-denatured ChABC | <0.001 | 5.005 | 15 |
| 3 | D | 10-25 μm :5xFAD-ChABC vs. 5xFAD-denatured ChABC | <0.001 | 10.75 | 15 |
| 3 | D | Below 10 μm :5xFAD-ChABC vs. 5xFAD-denatured ChABC | <0.001 | 16.05 | 15 |
| 3 | E | 5xFAD-ChABC vs. 5xFAD-denatured ChABC | <0.001 | 5.075 | 10 |
| 3 | G | 5xFAD-denatured ChABC vs. WT-denatured ChABC | <0.001 | 6.172 | 49.47 |
| 3 | G | 5xFAD-ChABC vs. 5xFAD-denatured ChABC | <0.001 | 4.45 | 66.08 |
| 3 | H | 5xFAD-denatured ChABC vs. WT-denatured ChABC | <0.001 | 4.443 | 40.47 |
| 3 | H | 5xFAD-ChABC vs. 5xFAD-denatured ChABC | 0.042 | 2.553 | 43.28 |
| 3 | I | 5xFAD-denatured ChABC vs. WT-denatured ChABC | <0.001 | 9.013 | 52.98 |
| 3 | I | 5xFAD-ChABC vs. 5xFAD-denatured ChABC | 0.538 | 1.215 | 63.05 |
| 3 | J | 5xFAD-denatured ChABC vs. WT-denatured ChABC | <0.001 | 5.424 | 30.1 |
| 3 | J | 5xFAD-ChABC vs. 5xFAD-denatured ChABC | 0.005 | 3.444 | 34.55 |
| 3 | L | 5xFAD-ChABC vs. 5xFAD-denatured ChABC | <0.001 |  |  |
| 3 | M | Below 30 μm: 5xFAD-ChABC vs. 5xFAD-denatured ChABC | 0.018 | 4.619 | 114 |
| 3 | M | 30-60 μm: 5xFAD-ChABC vs. 5xFAD-denatured ChABC | 0.039 | 4.234 | 114 |
| 3 | M | 60-90 μm: 5xFAD-ChABC vs. 5xFAD-denatured ChABC | <0.001 | 7.826 | 114 |
| 4 | B | WT-denatured ChABC vs. 5xFAD-denatured-ChABC | 0.58 | 1.364 | 15 |
| 4 | B | WT-denatured ChABC vs. 5xFAD-ChABC | 0.001 | 4.62 | 15 |
| 4 | B | 5xFAD-denatured-ChABC vs. 5xFAD-ChABC | 0.02 | 3.256 | 15 |
| 4 | C | WT-denatured ChABC vs. 5xFAD-denatured-ChABC | 0.25 | 1.864 | 15 |
| 4 | C | WT-denatured ChABC vs. 5xFAD-ChABC | 0.01 | 3.493 | 15 |
| 4 | C | 5xFAD-denatured-ChABC vs. 5xFAD-ChABC | <0.001 | 5.356 | 15 |
| 4 | D | WT-denatured ChABC vs. 5xFAD-denatured-ChABC | >0.99 | 0.9769 | 12 |
| 4 | D | WT-denatured ChABC vs. 5xFAD-ChABC | 0.004 | 4.171 | 12 |
| 4 | D | 5xFAD-denatured-ChABC vs. 5xFAD-ChABC | <0.001 | 5.148 | 12 |
| 4 | F | WT-denatured ChABC vs. 5xFAD-denatured ChABC | 0.05 | 2.566 | 26 |
| **Figure** | **Panel** | **Comparison** | **P-value** | **t (q)** | **DF(df)** |
| 4 | F | 5xFAD-denatured ChABC vs. 5xFAD-ChABC | 0.007 | 3.362 | 26 |
| 4 | G | WT-denatured ChABC vs. 5xFAD-denatured ChABC | 0.02 | 3.186 | 13 |
| 4 | G | WT-denatured ChABC vs. 5xFAD-ChABC | 0.17 | 2.084 | 13 |
| 4 | G | 5xFAD-denatured ChABC vs. 5xFAD-ChABC | <0.001 | 5.045 | 13 |
| 4 | I | ChABC vs. Denatured ChABC | 0.001 | 3.969 | 14 |
| 4 | J | ChABC vs. Denatured ChABC | 0.148 | 1.53 | 14 |
| 4 | K | ChABC vs. Denatured ChABC | 0.01 | 3.162 | 10 |
| 4 | L | ChABC vs. Denatured ChABC | 0.003 | 3.93 | 10 |
| 4 | M | EEA1: ChABC vs. Denatured ChABC | 0.262 | 1.304 | 4 |
| 4 | M | Rab7: ChABC vs. Denatured ChABC | 0.01 | 3.352 | 8 |
| 4 | M | Lamp1: ChABC vs. Denatured ChABC | 0.003 | 4.205 | 8 |
| 5 | E | WT-denatured ChABC vs. 5xFAD-denatured ChABC | 0.006 | 3.427 | 25 |
| 5 | E | WT-denatured ChABC vs. 5xFAD-ChABC | >0.99 | 0.3598 | 25 |
| 5 | E | 5xFAD-denatured ChABC vs. 5xFAD-ChABC | 0.003 | 3.691 | 25 |
| 5 | F | WT-denatured ChABC vs. 5xFAD-denatured ChABC | 0.03 | 2.341 | 25 |
| 5 | F | WT-denatured ChABC vs. 5xFAD-ChABC | 0.78 | 1.15 | 25 |
| 5 | F | 5xFAD-denatured ChABC vs. 5xFAD-ChABC | 0.01 | 2.943 | 25 |
| 5 | G | WT-denatured ChABC vs. 5xFAD-denatured ChABC | 0.04 | 2.208 | 21 |
| 5 | G | WT-denatured ChABC vs. 5xFAD-ChABC | 0.001 | 4.036 | 21 |
| 5 | G | 5xFAD-denatured ChABC vs. 5xFAD-ChABC | 0.03 | 2.352 | 21 |
| 5 | H | ChABC vs. Denatured ChABC | 0.002 | 3.365 | 32 |
| S1 | E | Mmp 12: 5xFAD vs. WT | <0.001 | 7.673 | 7 |
| S1 | E | Clec7a: 5xFAD vs. WT | <0.001 | 51.65 | 8 |
| S1 | E | Ccl3: 5xFAD vs. WT | <0.001 | 36.1 | 9 |
| S1 | E | Legals3: 5xFAD vs. WT | <0.001 | 12.72 | 10 |
| S1 | E | Tgm1: 5xFAD vs. WT | <0.001 | 3.193 | 10 |
| S1 | E | CtsE: 5xFAD vs. WT | <0.001 | 9.95 | 7 |
| S1 | E | Optc: 5xFAD vs. WT | <0.001 | 5.104 | 8 |
| S1 | E | Cxcl11: 5xFAD vs. WT | <0.001 | 8.604 | 6 |
| S1 | E | Col19a1: 5xFAD vs. WT | <0.001 | 9.153 | 10 |
| S1 | E | Col4a3: 5xFAD vs. WT | <0.001 | 10.63 | 4 |
| S1 | E | Pappa2: 5xFAD vs. WT | <0.001 | 12.27 | 6 |
| S1 | F | 5xFAD-denatured ChABC vs. 5xFAD-ChABC | <0.001 | 7.608 | 20 |
| S2 | B | Adam5 | 0.027 | 2.707 | 8 |
| S2 | B | Adamts1 | <0.001 | 35.31 | 8 |
| S2 | B | Adamts9 | <0.001 | 12.08 | 8 |
| S2 | B | Adamts14 | 0.014 | 3.21 | 7 |
| S2 | B | Mmp3 | 0.004 | 3.991 | 8 |
| S2 | B | Mmp25 | <0.001 | 5.187 | 8 |
| S2 | D | Csf3: WT-denatured ChABC vs. 5xFAD-denatured ChABC | <0.001 | 11.09 | 7 |
| S2 | D | Csf3: 5xFAD-denatured ChABC vs. 5xFAD-ChABC | <0.001 | 12.29 | 7 |
| S2 | D | Clec4a1: WT-denatured ChABC vs. 5xFAD-denatured ChABC | 0.611 | 1.381 | 8 |
| S2 | D | Clec4a1: 5xFAD-denatured ChABC vs. 5xFAD-ChABC | <0.001 | 34.07 | 8 |
| **Figure** | **Panel** | **Comparison** | **P-value** | **t (q)** | **DF(df)** |
| S2 | D | Tnfsf8: 5xFAD-denatured ChABC vs. 5xFAD-ChABC | <0.001 | 31.26 | 8 |
| S2 | D | Mmp3: WT-denatured ChABC vs. 5xFAD-denatured ChABC | 0.027 | 3.413 | 6 |
| S2 | D | Mmp3: 5xFAD-denatured ChABC vs. 5xFAD-ChABC | <0.001 | 67.6 | 6 |
| S2 | D | Adamts1: WT-denatured ChABC vs. 5xFAD-denatured ChABC | 0.99 | 0.1932 | 9 |
| S2 | D | Adamts1: 5xFAD-denatured ChABC vs. 5xFAD-ChABC | <0.001 | 56.5 | 9 |
| S2 | D | Ccl5: WT-denatured ChABC vs. 5xFAD-denatured ChABC | 0.015 | 5.073 | 9 |
| S2 | D | Ccl5: 5xFAD-denatured ChABC vs. 5xFAD-ChABC | <0.001 | 125.6 | 9 |
| S2 | D | Clec4e: WT-denatured ChABC vs. 5xFAD-denatured ChABC | 0.004 | 6.326 | 9 |
| S2 | D | Clec4e: 5xFAD-denatured ChABC vs. 5xFAD-ChABC | <0.001 | 19.27 | 9 |
| S3 | A | 5xFAD-denatured ChABC vs. 5xFAD-ChABC | <0.001 | 5.797 | 34 |
| S3 | D | WT-denatured ChABC vs. 5xFAD-denatured ChABC | 0.004 | 3.976 | 15 |
| S3 | D | WT-denatured ChABC vs. 5xFAD-ChABC | 0.81 | 1.146 | 15 |
| S3 | D | 5xFAD-denatured ChABC vs. 5xFAD-ChABC | 0.04 | 2.831 | 15 |
| S4 | A | WT-denatured ChABC vs. WT-ChABC | 0.912 | 0.9331 | 44 |
| S4 | A | WT-denatured ChABC vs. 5xFAD-denatured ChABC | 0.87 | 1.082 | 44 |
| S4 | A | 5xFAD-denatured ChABC vs. 5xFAD-ChABC | 0.929 | 0.8585 | 44 |
| S4 | B | WT-denatured ChABC vs. WT-ChABC | >0.999 | 1.102 | 44 |
| S4 | B | WT-denatured ChABC vs. 5xFAD-denatured ChABC | >0.999 | 1.056 | 44 |
| S4 | B | 5xFAD-denatured ChABC vs. 5xFAD-ChABC | >0.999 | 1.037 | 44 |
| S4 | C | WT-denatured ChABC vs. WT-ChABC | 0.96 | 0.0537 | 22 |
| S4 | D | WT-denatured ChABC vs. WT-ChABC | 0.99 | 0.0141 | 22 |
| S4 | E | WT-denatured ChABC vs. WT-ChABC | 0.62 | 0.5059 | 22 |
| S5 | B | WT-denatured ChABC vs. WT-ChABC | 0.977 | 0.5729 | 15 |
| S5 | B | WT-denatured ChABC vs. 5xFAD-denatured ChABC | <0.001 | 7.796 | 15 |
| S5 | B | 5xFAD-denatured ChABC vs. 5xFAD-ChABC | 0.992 | 0.3928 | 15 |
| S5 | C | WT-denatured ChABC vs. WT-ChABC | >0.999 | 0.4245 | 20 |
| S5 | C | WT-denatured ChABC vs. 5xFAD-denatured ChABC | 0.011 | 3.608 | 20 |
| S5 | C | 5xFAD-denatured ChABC vs. 5xFAD-ChABC | >0.999 | 0.4245 | 20 |
| S5 | D | WT-denatured ChABC vs. WT-ChABC | >0.999 | 0.4218 | 20 |
| S5 | D | WT-denatured ChABC vs. 5xFAD-denatured ChABC | <0.001 | 5.589 | 20 |
| S5 | D | 5xFAD-denatured ChABC vs. 5xFAD-ChABC | >0.999 | 0.5624 | 20 |
| S5 | E | WT-denatured ChABC vs. WT-ChABC | >0.999 | 0.4935 | 20 |
| S5 | E | WT-denatured ChABC vs. 5xFAD-denatured ChABC | >0.999 | 0.7488 | 20 |
| S5 | E | 5xFAD-denatured ChABC vs. 5xFAD-ChABC | >0.999 | 0.6691 | 20 |
| S6 | B | WT- denatured ChABC vs. WT-ChABC | 0.49 | 0.7172 | 20 |
| S6 | B | WT- denatured ChABC vs. 5xFAD- denatured ChABC | 0.039 | 2.37 | 20 |
| S6 | B | 5xFAD- denatured ChABC vs. 5xFAD-ChABC | 0.109 | 1.761 | 20 |
| S6 | C | WT- denatured ChABC vs. WT-ChABC | 0.764 | 0.3084 | 20 |
| S6 | C | WT- denatured ChABC vs. 5xFAD-denatured ChABC | <0.001 | 5.52 | 20 |
| S6 | C | 5xFAD- denatured ChABC vs. 5xFAD-ChABC | 0.48 | 0.7316 | 20 |
| S6 | D | WT-denatured ChABC vs. WT-ChABC | >0.999 | 0.3081 | 20 |
| S6 | D | WT-denatured ChABC vs. 5xFAD-denatured ChABC | >0.999 | 0.06754 | 20 |
| S6 | D | 5xFAD-denatured ChABC vs. 5xFAD-ChABC | >0.999 | 0.9846 | 20 |
| **Figure** | **Panel** | **Comparison** | **P-value** | **t (q)** | **DF(df)** |
| S7 | C | WT-denatured ChABC vs. 5xFAD-denatured ChABC | 0.006 | 4.29 | 9 |
| S7 | C | 5xFAD-denatured ChABC vs. 5xFAD-ChABC | >0.99 | 0.59 | 9 |
| S7 | D | WT-denatured ChABC vs. 5xFAD-denatured ChABC | <0.001 | 5.636 | 9 |
| S7 | D | 5xFAD-denatured ChABC vs. 5xFAD-ChABC | 0.9 | 1.104 | 9 |
| S7 | E | WT-denatured ChABC vs. 5xFAD-denatured ChABC | 0.02 | 3.551 | 9 |
| S7 | E | 5xFAD-denatured ChABC vs. 5xFAD-ChABC | 0.71 | 1.266 | 9 |
| S7 | F | *Ide* | 0.084 | 2.531 | 18 |
| S7 | F | *Ace* | >0.999 | 0.2488 | 18 |
| S7 | F | *tPA* | 0.22 | 2.052 | 18 |
| S7 | F | *NeP* | 0.168 | 2.189 | 18 |
| S7 | G | 5xFAD-denatured ChABC vs. WT-denatured ChABC | <0.001 | 5.017 | 34.68 |
| S7 | G | 5xFAD-ChABC vs. 5xFAD-denatured ChABC | >0.999 | 1.366 | 566.9 |
| S7 | H | 5xFAD-denatured ChABC vs. WT-denatured ChABC | <0.001 | 8.326 | 70.33 |
| S7 | H | 5xFAD-ChABC vs. 5xFAD-denatured ChABC | >0.999 | 2.371 | 168.8 |
| S7 | I | 5xFAD-denatured ChABC vs. WT-denatured ChABC | <0.001 | 4.588 | 85.77 |
| S7 | I | 5xFAD-ChABC vs. 5xFAD-denatured ChABC | <0.001 | 2.966 | 1270 |
| S7 | J | 5xFAD-denatured ChABC vs. WT-denatured ChABC | <0.001 | 11.43 | 73.99 |
| S7 | J | 5xFAD-ChABC vs. 5xFAD-denatured ChABC | 0.12 | 2.06 | 101.5 |
| S8 | A | WT-denatured ChABC vs. 5xFAD-denatured ChABC | 0.505 |  |  |
| S8 | E | ChABC(-)Oligomer(+) vs. ChABC(+)Oligomer(+) | <0.001 | 7.643 | 68 |
| S8 | E | ChABC(-)Oligomer(+) vs. ChABC(-)Polymer(+) | 0.7 | 1.59 | 68 |
| S8 | E | ChABC(+)Oligomer(+) vs. ChABC(+)Polymer(+) | >0.999 | 0.4302 | 68 |
| S8 | E | ChABC(-)Polymer(+) vs. ChABC(+)Polymer(+) | <0.001 | 11.26 | 68 |
| S8 | G | ChABC(-)Oligomer(+) vs. ChABC(+)Oligomer(+) | >0.999 | 0.2508 | 84 |
| S8 | G | ChABC(-)Oligomer(+) vs. ChABC(-)Polymer(+) | 0.011 | 3.209 | 84 |
| S8 | G | ChABC(+)Oligomer(+) vs. ChABC(+)Polymer(+) | >0.999 | 0.2924 | 84 |
| S8 | G | ChABC(-)Polymer(+) vs. ChABC(+)Polymer(+) | 0.112 | 2.4 | 84 |
| S9 | B | ChABC vs. Denatured ChABC | <0.001 | 4.357 | 35 |
| S9 | D | GFP^-^mCherry^+^: ChABC vs. Control | 0.01 | 2.966 | 26 |
| S9 | D | GFP^+^mCherry^+^: ChABC vs. Control | 0.008 | 3.148 | 26 |
| S10 | A | ChABC vs. Denatured ChABC | 0.056 | 2.086 | 14 |
| S10 | B | ChABC vs. Denatured ChABC | 0.007 | 3.371 | 10 |
| S10 | C | ChABC vs. Denatured ChABC | 0.029 | 2.555 | 10 |
| S10 | D | Control vs. ChABC | <0.001 | 3.631 | 91 |
| S10 | D | Control vs. BafA1 | 0.005 | 2.972 | 91 |
| S10 | D | Control vs. BafA1+ ChABC | 0.248 | 1.167 | 91 |
| S10 | D | BafA1 vs. BafA1+ ChABC | 0.058 | 1.964 | 91 |
| S11 |  | *Cd74* | <0.001 | 7.197 | 12 |
| S11 |  | *Rab27a* | 0.02 | 2.731 | 12 |
| S11 |  | *Tlr1* | <0.001 | 5.664 | 12 |
| S11 |  | *Cybb* | <0.001 | 9.859 | 12 |
| S11 |  | *Myo1g* | <0.001 | 7.929 | 12 |
| S11 |  | *Arhgap25* | 0.004 | 3.54 | 12 |
| **Figure** | **Panel** | **Comparison** | **P-value** | **t (q)** | **DF(df)** |
| S11 |  | *Bin2* | <0.001 | 5.619 | 12 |
| S11 |  | *Lcp1* | <0.001 | 8.845 | 12 |
| S12 | A | WT-denatured ChABC vs. 5xFAD-ChABC | 0.385 | 1.61 | 15 |
| S12 | A | 5xFAD-denatured ChABC vs. 5xFAD-ChABC | 0.408 | 1.576 | 15 |
| S12 | B | 5xFAD-denatured ChABC vs. 5xFAD-ChABC | 0.765 | 0.3028 | 22 |
| S12 | C | 5xFAD-denatured ChABC vs. 5xFAD-ChABC | 0.36 | 0.9397 | 18 |

**Data S1. GO enrichment of differential expression proteins in WT mice under ChABC treatment.**

**Data S2. The GO enrichments of differentially expressed genes in PFC in WT and 5xFAD mice**

**Data S3. The TMT-MS data in PFC from 5xFAD mice under ECM remodeling**

**Data S4. RNAseq data of astrocytes from PFC of 5xFAD mice under ECM remodeling**
